# Near-infrared electroluminescence beyond 940 nm in Pt(N^C^N)X complexes: influencing aggregation with the ancillary ligand X†

**DOI:** 10.1039/d2sc05023d

**Published:** 2022-11-11

**Authors:** Rebecca J. Salthouse, Piotr Pander, Dmitry S. Yufit, Fernando B. Dias, J. A. Gareth Williams

**Affiliations:** Department of Chemistry, Durham University South Road Durham DH1 3LE UK j.a.g.williams@durham.ac.uk; Faculty of Chemistry, Silesian University of Technology M. Strzody 9 Gliwice 44-100 Poland piotr.pander@polsl.pl; Department of Physics, Durham University South Road Durham DH1 3LE UK f.m.b.dias@durham.ac.uk

## Abstract

We present a study of aggregate excited states formed by complexes of the type Pt(N^C^N)X, where N^C^N represents a tridentate cyclometallating ligand, and X = SCN or I. These materials display near-infrared (NIR) photoluminescence in film and electroluminescence in NIR OLEDs with *λ*^max^_EL_ = 720–944 nm. We demonstrate that the use of X = SCN or I modulates aggregate formation compared to the parent complexes where X = Cl. While the identity of the monodentate ligand affects the energy of Pt–Pt excimers in solution in only a subtle way, it strongly influences aggregation in film. Detailed calculations on aggregates of different sizes support the experimental conclusions from steady-state and time-resolved luminescence studies at variable temperatures. The use of X = I appears to limit aggregation to the formation of dimers, while X = SCN promotes the formation of larger aggregates, such as tetramers and pentamers, leading in turn to NIR photo- and electroluminescence > 850 nm. A possible explanation for the contrasting influence of the monodentate ligands is the lesser steric hindrance associated with the SCN group compared to the bulkier I ligand. By exploiting the propensity of the SCN complexes to form extended aggregates, we have prepared an NIR-emitting OLED that shows very long wavelength electroluminescence, with *λ*^max^_EL_ = 944 nm and a maximum EQE = 0.3 ± 0.1%. Such data appear to be unprecedented for a device relying on a Pt(ii) complex aggregate as the emitter.

## Abstract 2

The ancillary ligand X strongly influences the aggregates formed by complexes of the type Pt(N^C^N)X. X = I leads to a 4× increase in film PLQY and OLED EQE *vs.* X = Cl, while X = SCN yields very long wavelength electroluminescence, *λ*^max^_EL_ = 944 nm.

## Graphical abstract

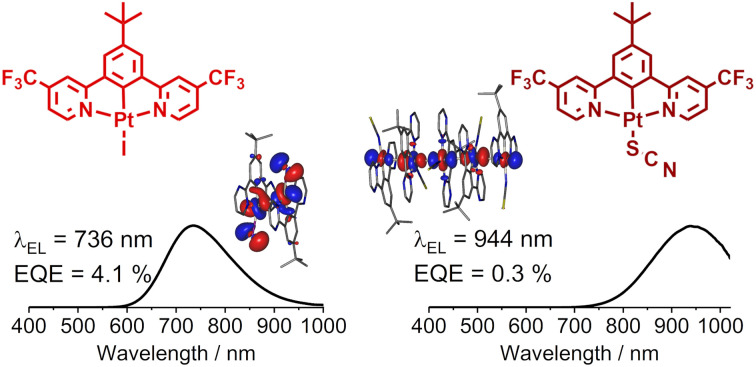

## Introduction

Near-infrared (NIR) photo- and electroluminescence is desirable for many applications in modern technology, including night vision, communications,^1^ and security applications.^2^ The NIR emission range (700–1400 nm) coincides with a “window of transparency” of biological tissue – a region where few endogenous biological molecules absorb. NIR radiation can thus penetrate deeply into tissue, and NIR organic light-emitting diodes (OLEDs) have potential in a variety of biomedical and bio-sensing applications, including bio-imaging *in vivo* and photodynamic therapy (PDT).^3–6^

OLED emitters based on organometallic complexes have attracted much attention due to their stability, ease of colour tuning, and high photoluminescence efficiencies that arise from triplet as well as singlet states leading to emission.^7^ Whilst iridium(iii) complexes remain the usual choice^8^ for visible-light displays, planar platinum(ii) complexes that form aggregates through face-to-face interactions offer attractions in NIR OLEDs.^9–18^ These properties are unique to square-planar complexes of d^8^ metal ions, arising from the interaction between d_*z*^2^_ and p_*z*_ orbitals of the metal centres that are orthogonal to the plane.^19–23^ These interfacial interactions, which may also involve π–π interactions between aromatic ligands, may lead to the formation of excimers, dimers and larger aggregates (oligomers) in solution and solid state. Excited states originating from these species {*e.g.*, those of metal–metal-to-ligand charge-transfer (MMLCT) character^11,24,25^} tend to be lower in energy than the isolated molecules, often leading to characteristic structureless emission that may extend deep into the red and NIR regions.^26^ Despite the challenges in generating efficient NIR emission associated with the energy-gap law,^27^ the highest reported PLQY of a thin-film Pt(ii) complex to date has reached 81% with λ^max^_PL_ = 740 nm, in work by Chi and colleagues.^9^ Exciton-like emission along the molecular aggregate and the d_*z*^2^_ orbital suppresses exciton-optical phonon coupling, and OLEDs fabricated with the 2-pyrazinyl pyrazolate Pt(ii) complex showed external quantum efficiencies (EQEs) of 24%.

Numerous designs of Pt(ii) complexes have been considered for OLEDs, according to the nature of the ligands around the metal centre.^13,17,28^ Those involving tridentate, N^C^N-coordinating ligands^29^ have proved to be particularly attractive in terms of emission efficiency, colour tuning, and generation of red/NIR emission through intermolecular interactions. They do require an ancillary monodentate ligand X to complete the coordination sphere around the Pt(ii) centre. Most synthetic procedures lead to the compounds where X = Cl, but X can easily be exchanged for other monoanionic ligands such as thiolates (RS^−^),^30^ acetylides (RC≡C^−^),^31,32^ or other halogen and pseudohalogen anions including iodide,^33^ cyanide (CN^−^),^34^ and thiocyanate (SCN^−^).^35^ With the exception of RS^−^, the effect on the solution emission is usually quite limited, especially the emission wavelength region. However, some of the current authors have demonstrated that the change from X = Cl to NCS can lead to a substantial red-shift of the luminescence in neat films.^35^ We have also observed that complexes with X = I form lower-energy excimers than those with X = Cl.^33^ These concepts of ancillary ligand exchange form the basis of this work.

**Fig. 1:**
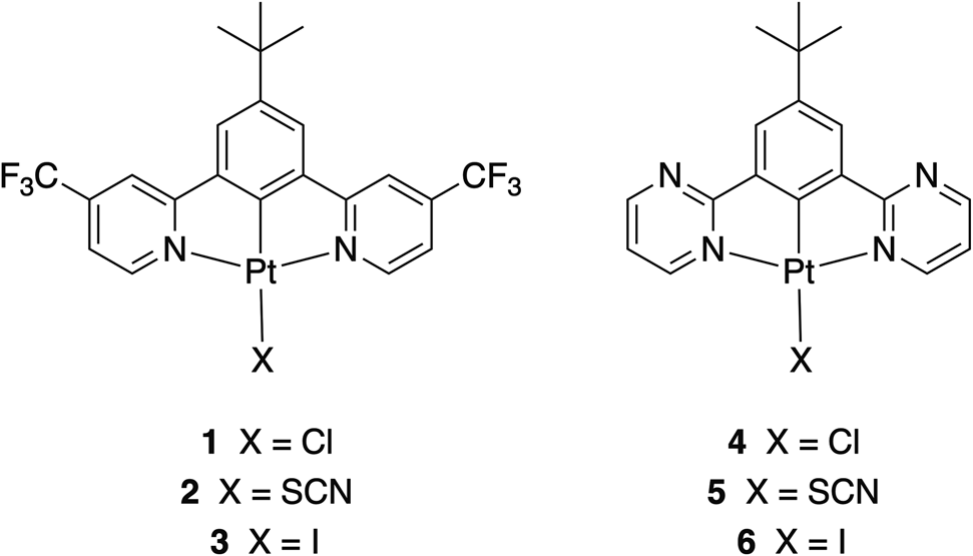
Structures of the complexes 2, 3, 5, and 6 prepared and studied in this work, together with those of the recently reported parent complexes 1 and 4 with X = Cl.^36^

This study follows on from recent work in which we investigated the aggregation of complexes 1 and 4, and the use of very thin layers of these materials to generate NIR OLEDs.^36^ The selection of complexes 1 and 4 in that work was made on the basis that the electron-withdrawing nature of the CF_3_ groups in 1, and the electron-deficient nature of the pyrimidine rings in 4, leads to stabilisation of the LUMO and a shift in the emission towards the red/NIR (Fig. 1). In the present work, we show that the corresponding X = I derivatives (3 and 6) display a four-fold increase in NIR OLED EQE and film PLQY, while maintaining a similar *λ*_EL_ of 740 nm. Moreover, we show that for the thiocyanate derivatives (2 and 5), the NIR electroluminescence is shifted to *λ*^max^_EL_ > 940 nm, a value that appears to be the longest yet reported for aggregated Pt(ii) complexes. Indeed, amongst platinum-based complexes, only those of π-extended benzoporphyrins emit further into the NIR.^37^

## Target complexes and their synthesis

Complexes 1 and 4 were selected as parent structures owing to their excellent performance in NIR-OLEDs.^36^ They were synthesised as previously reported in that work. The subsequent metathesis of the chloride ligand was achieved by treatment firstly with silver trifluoromethanesulfonate (AgOTf) in acetone solution at room temperature. This process leads to the removal of the chloride ligand through precipitation of AgCl, and its replacement by acetone as a weakly-bound ligand in the fourth site. Addition of KI or KNCS then leads to the replacement of the acetone ligand by I^−^ or SCN^−^ to give the desired products (2, 3, 5 and 6). The products were purified by a series of washings and recrystallisation, as described in the ESI.†

Thermogravimetric analysis (TGA) of the complexes 2, 3, 5, and 6 demonstrates their high thermal stability (Fig. S2.7†). The temperature at which a 5% weight loss was recorded exceeds 290 °C in all cases: complex 2, 318 °C; 3, 305 °C; 5, 295 °C, and 6, 312 °C.

Crystals of complex 6 suitable for X-ray diffraction analysis were grown from a solution in chloroform, and its structure determined accordingly (Fig. S3.2†). Attempts to obtain crystals of 2 were successful only in CHCl_3_, in which solvent some transformation to 1 occurs over the prolonged periods required for crystallisation (probably due to trace HCl), leading to SCN/Cl disorder in the structure. Nevertheless, the molecular structure of 2 (Fig. S3.1†) reveals that the thiocyanate ligand is bound through the sulphur atom (*i.e.*, thiocyanate not isothiocyanate), in contrast with the previous literature report of a Pt(N^C^N)NCS complex, where the ligand was bound through nitrogen.^35^ The planar structures are quite typical of Pt(dpyb)Cl and other Pt(N^C^N)X complexes that have been structurally characterised, with short Pt–C bonds averaging 1.939 and 1.905 Å for 2 and 6 respectively, and N–Pt–N angles of 161 and 160° respectively. Despite the relative lability of the Pt–SCN bond evident from the decomposition in CHCl_3_, the materials are stable indefinitely in the solid state. The molecules of 2 pack in a head-to-tail arrangement with a closest Pt⋯Pt distance of 5.3560(9) Å that implies the absence of metallophilic interactions, at least in this particular crystal, but the shortest interplanar distance is 3.484(7) Å, suggestive of some weak π–π interactions at play. Similar conclusions are drawn for complex 6, where the corresponding distances are 5.0619(5) Å and 3.4634(8) Å.

## Solution-state photophysics

### Steady-state absorption and emission spectra

**Fig. 2:**
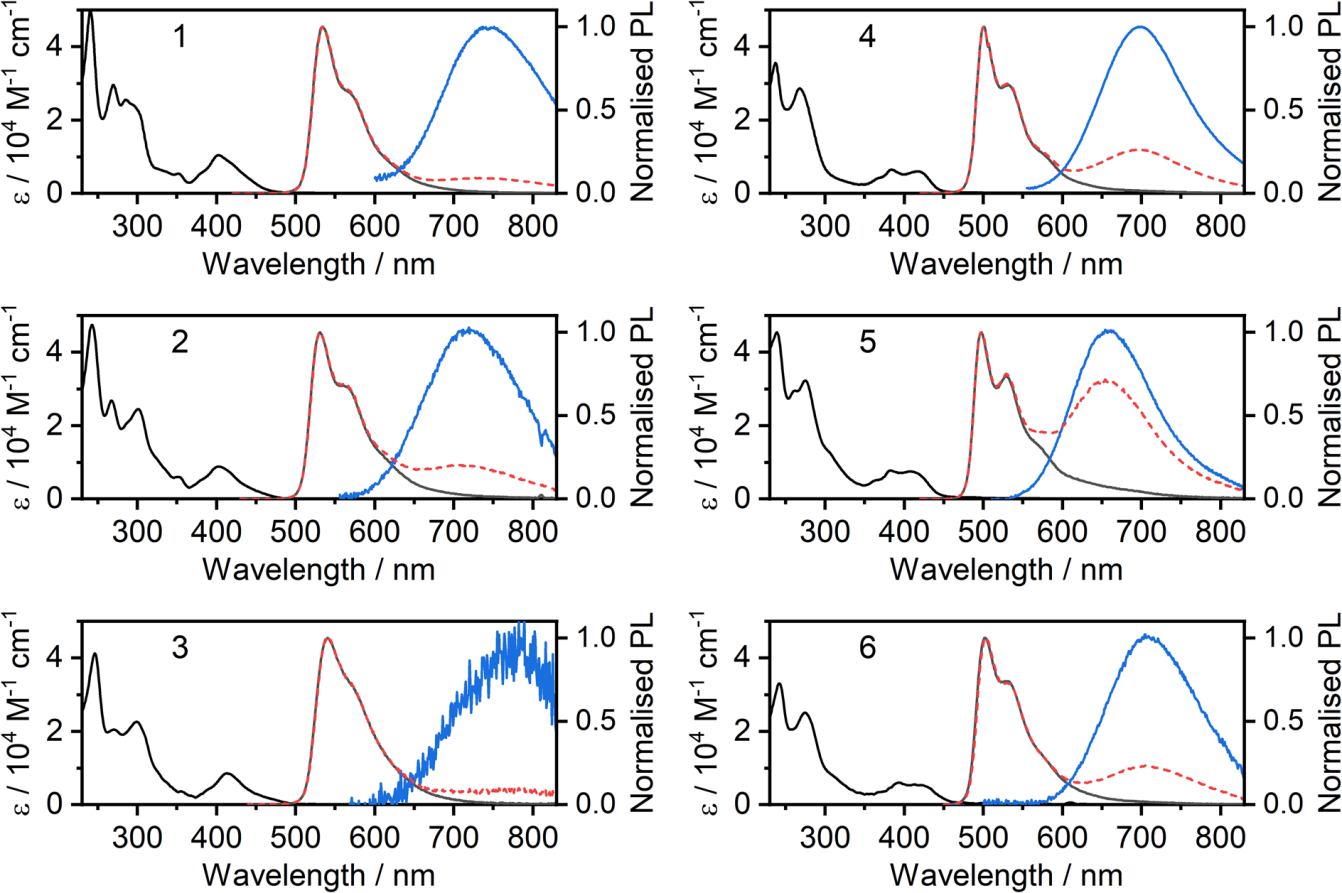
Photoluminescence of complexes 2, 3, 5, and 6 in CH_2_Cl_2_ solutions: absorption (black lines) and photoluminescence (grey) spectra in dilute (≈10^−5^ M) solution; photoluminescence spectra at higher concentration (≈10^−4^ M, dashed red); deconvoluted excimer spectrum (blue).

The absorption and photoluminescence spectra of the new complexes 2, 3, 5 and 6 in CH_2_Cl_2_ solution at room temperature are shown in Fig. 2, together with those of the parents 1 and 4 for comparison. A summary of all spectroscopic data is shown in Table 1, while a breakdown of remaining key luminescence properties for all complexes is given in the ESI (Table S8.1†). It is apparent that the ancillary ligand has relatively little effect on the absorption and unimolecular emission: the spectra for 2 and 3 are similar to those of 1, whilst 5 and 6 are similar to 4. Nevertheless, there are some differences. The X = I complexes display slightly red-shifted photoluminescence compared to X = SCN, and a small red-shift of the lowest energy absorption band. The most remarkable results to note are the high *Φ*_PL_ values, close to unity for complexes 2 and 3. The overall *Φ*_PL_ decreases as the concentration is increased, due to self-quenching of the unimolecular photoluminescence and the lower luminescence yield of the resulting excimer (Fig. S5.27†).

**Table 1:** Photophysical data for complexes 2, 3, 5 and 6 in deoxygenated CH_2_Cl_2_ solution at room temperature, together with figures for the recently reported parent complexes 1 and 4 with X = Cl^36^

	*λ*_abs_/nm (*ε*/M^−1^ cm^−1^)	*λ*_em_ a/nm unimol	*λ*_em_ b/nm excimer	*Φ*_PL_ c	*τ*_0_ d/μs	*k*_r_ e/10^5^ s^−1^	*k*_nr_ e/10^5^ s^−1^	*k*_SQ_ f/10^9^ M^−1^ s^−1^
1	244 (36 200), 272 (23 000), 290 (19 800), 305 (16 700), 358 (3460), 382sh (4250), 405 (7630)	533, 570	750	0.91	6.5	1.4	0.1	1.0
2	245 (45 800), 269 (26 100), 305 (23 000), 356 (5400), 405 (8700)	531, 567	720	1.0	7.3	1.9	—	1.4
3	248 (38 800), 274 (19 700), 304 (20 800), 360 (3520), 415 (8300)	541, 573	∼775	0.92	6.1	1.6	0.1	2.0
4	240 (26 086), 270 (22 070), 362sh (2550), 387 (4833), 420 (4484)	500, 531, 584sh	701	0.60	7.9	0.81	0.5	1.5
5	266 (35 500), 279 (29 000), 359 (4120), 388 (7400), 400 (7180), 417 (7080)	497	659	0.67	8.4	1.0	0.5	2.9
6	244 (32 276), 279 (23 125), 400 (4976), 420 (5012)	502	709	0.86	6.6	1.4	0.2	2.4

a *λ*_max_ value for unimolecular emission bands.

b *λ*_max_ value for the excimer band.

c Luminescence quantum yield in dilute deoxygenated solution, measured using [Ru(bpy)_3_]Cl_2_ (aq) as the standard (*Φ*_PL_ = 0.04).^38^ Self-quenching is negligible under these dilute conditions, such that the *Φ*_PL_ values refer specifically to the unimolecular photoluminescence.

d Lifetime at infinite dilution, estimated by extrapolation of a plot of 1/*τ versus* concentration, *c*, to *c* = 0.

e Estimates of *k*_r_ and *k*_nr_ assuming that the emitting state is formed with unit efficiency such that *k*_r_ = *Φ*/*t* and *k*_nr_ = (1 − *Φ*)/*τ*.

f Self-quenching rate constant estimated from the gradient of the plot of 1/*τ versus* concentration.

At higher concentrations, all four complexes display excimer formation (Fig. 2 and S5.1–S5.3†), manifested by the emergence of a new broad band in the luminescence spectrum with *λ*_max_ in the range 650 to 775 nm. Such behaviour is typical of many other Pt(N^C^N)X complexes. In the pyrimidine series, the change from X = Cl to I (4 to 6) is accompanied by only a very marginal red-shift in the excimer emission. For the CF_3_-substituted pyridine series, the excimer of the iodo derivative 3 is red-shifted rather more to *λ*^max^ ∼775 nm. However, the intensity of this long-wavelength excimer band is very low, even at elevated concentration, and we have therefore not probed it further.

**Fig. 3:**
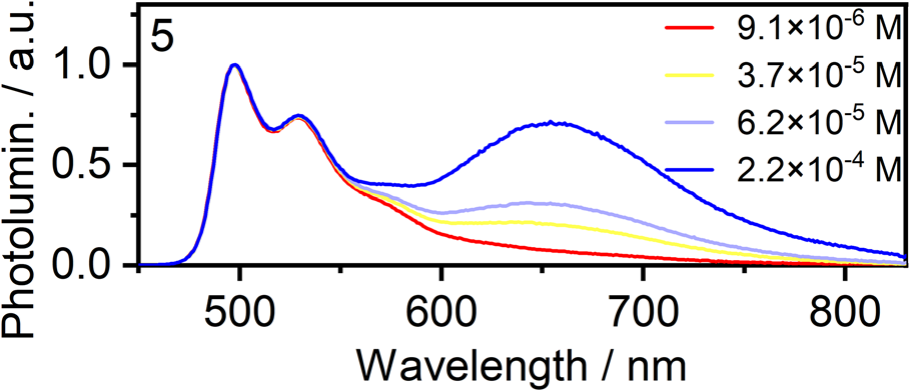
Normalised photoluminescence spectra of complex 5 in CH_2_Cl_2_ at various concentrations indicated in the legend.

Concentration-dependent emission studies reveal that complex 5 has the highest propensity to excimer formation among the newly synthesized complexes as its self-quenching constant is the largest (Table 1 and Fig. 3). The ancillary ligand is clearly playing a role in the intermolecular interactions and thus excimer emission: lower energy excimers are observed for X = I and higher energy for X = –NCS/SCN.

## Solid-state photophysics

**Fig. 4:**
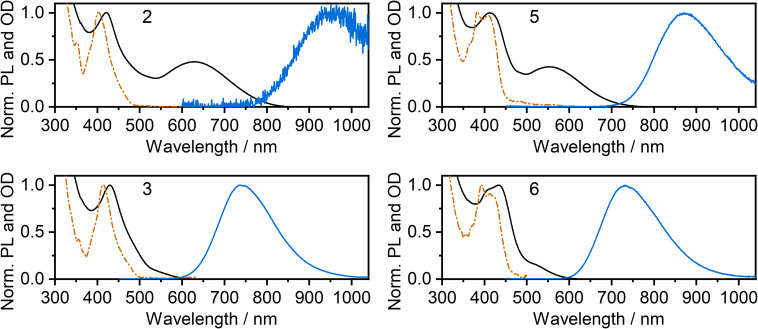
Photoluminescence (blue lines) and absorption spectra (black lines) of complexes 2, 3, 5, and 6 in thermally evaporated neat films. Absorption spectra in dilute CH_2_Cl_2_ solution (orange dashed and dotted line) from Fig. 2 are reproduced for comparison.

The photoluminescence and absorption spectra of complexes 2, 3, 5 and 6 in neat films are presented in Fig. 4; the low-energy portion of the corresponding absorption spectrum in dilute CH_2_Cl_2_ solution is shown for comparison (reproduced from Fig. 2). In each case, the absorption band in film at around 400 to 450 nm is similar to that recorded in solution and the band can thus be attributed to absorption of light by individual molecules. However, in the long wavelength region, the spectra in neat film and solution show a striking difference from one another: an additional low-energy absorption band is present for the film. For the iodo complexes 3 and 6, this low-energy band appears as a weak shoulder at around 500–600 nm, but it is much more pronounced for the NCS/SCN complexes 2 and 5, forming a clear maximum at *λ* = 629 and 552 nm respectively. Previous studies,^9,11,39^ as well as computations presented in this work below, suggest that this long wavelength band can be attributed to metal–metal-to-ligand charge-transfer (MMLCT) transitions. All four complexes display NIR luminescence in neat films (as opposed to the structured green emission observed in dilute solution). Thus, it is evident that the observed NIR luminescence in film originates from excited states of aggregates formed in the film, probably of MMLCT nature. The *λ*_max_ values of the PL of 3 and 6 in film (740 and 733 nm respectively) are similar to those of the respective excimers in solution (Fig. 2). However, for the NCS/SCN complexes 2 and 5, the *λ*_max_ values in the film of 950 and 872 nm, respectively, are greatly shifted compared to those of the excimer in solution (720 and 659 nm respectively).

**Fig. 5:**
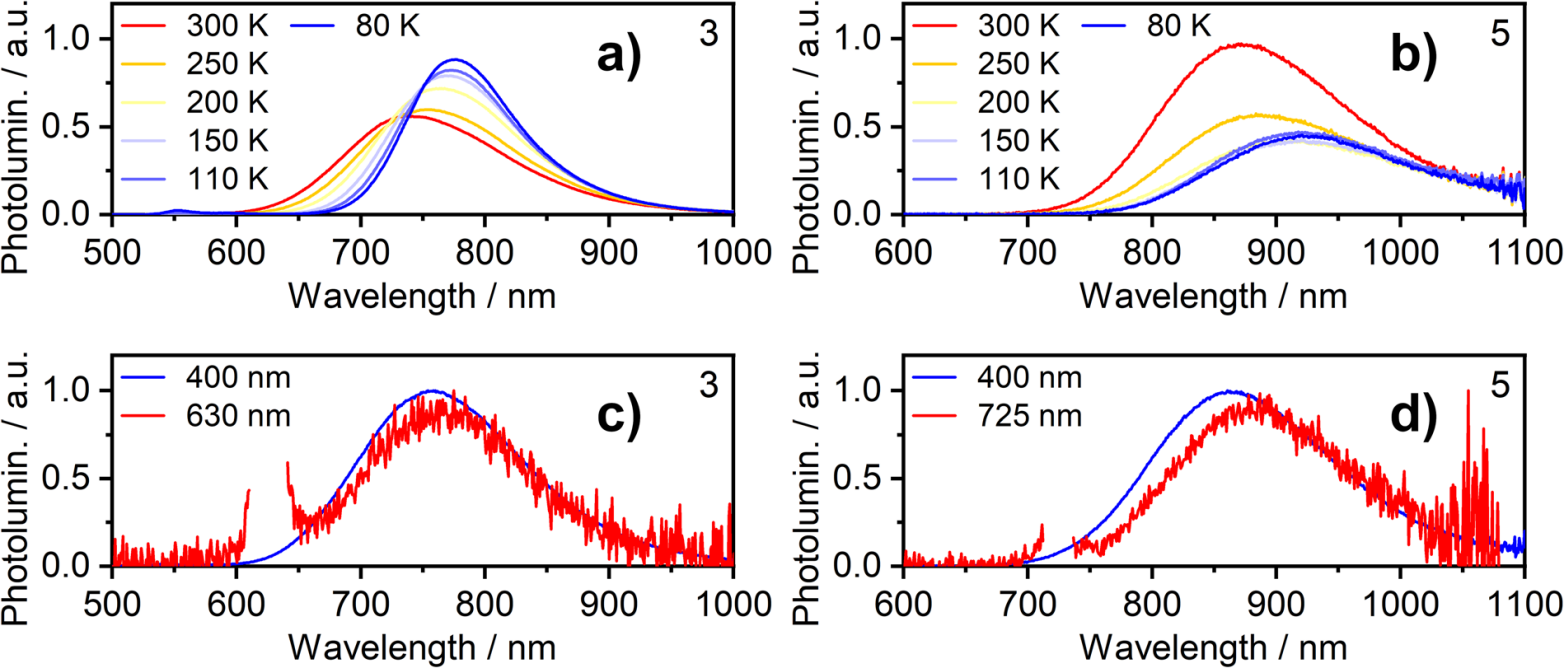
Photoluminescence of complexes 3 and 5 in neat thermally deposited films: (a and b) PL spectra recorded at temperatures from 300 K to 80 K with 365 nm excitation wavelength; (c and d) PL spectra recorded at room temperature with long and short wavelength excitation as indicated in figure legend.

Further insight into the properties of 2, 3, 5 and 6 in film was obtained by examining the photoluminescence spectra recorded using a range of excitation wavelengths and at variable temperature (Fig. 5 for 3 and 5; S5.4–S5.19† for 2 and 6). The PL spectrum becomes subtly red-shifted when longer wavelength excitation is used (though the trend is very small for 3). This observation suggests that more than one “type” of aggregate is present in the film and that the PL from higher-energy aggregate excited states (expected to be associated with the smallest aggregates, for example, dimers) contribute significantly less to the luminescence spectrum when using excitation wavelengths close to the absorption onset. These conclusions are supported by time-resolved measurements (Fig. S5.20–5.26†). For example, for neat films of 5 and, more clearly, 6, an initial rise in intensity is evident in the decay traces recorded at longer emission wavelengths (*e.g. λ*_em_ = 800 nm for 6) with a corresponding quenching of shorter-wavelength luminescence (*e.g. λ*_em_ = 680 nm) over the same timescale of around 20–100 ns, which is consistent with energy transfer processes taking place between different luminescent species (such as aggregates of different sizes).

The temperature dependence of the emission from neat films of complexes 2, 3, 5 and 6 falls into one of two types of behaviour. (1) For 2, 3 and 5, *λ*_max_ red-shifts quite significantly with decreasing temperature, accompanied by a moderate increase (2 and 3) or decrease (5) of the total PL intensity (the shift and intensity change for 2 are small but the very low intensity in this case engenders more uncertainty). (2) For 6, *λ*_max_ scarcely changes, but the PL intensity substantially increases. Both classes of behaviour have been observed in our previous work on the parent chloro complexes {1 displays behaviour (2), while 4 shows behaviour (1)}. We concluded that in case (1), the PL spectrum is probably dominated by larger aggregates at low temperature – either by energy transfer to them from smaller aggregates or due to larger aggregates being formed at lower temperatures; such appears to be the case for 2, 3, and 5. In case (2) shown by 6, on the other hand, it appears that the same emissive aggregate species remains dominant at low temperatures but, due to reduced molecular vibrations, the PL spectrum narrows and its intensity increases.

The photoluminescence decay in film follows mono-exponential kinetics in all cases at RT and mostly remains so at lower temperatures. The observed decay lifetime increases at lower temperature, consistent with suppressed non-radiative decay under these conditions (Tables S5.1–5.4†).

## Calculations

**Fig. 6:**
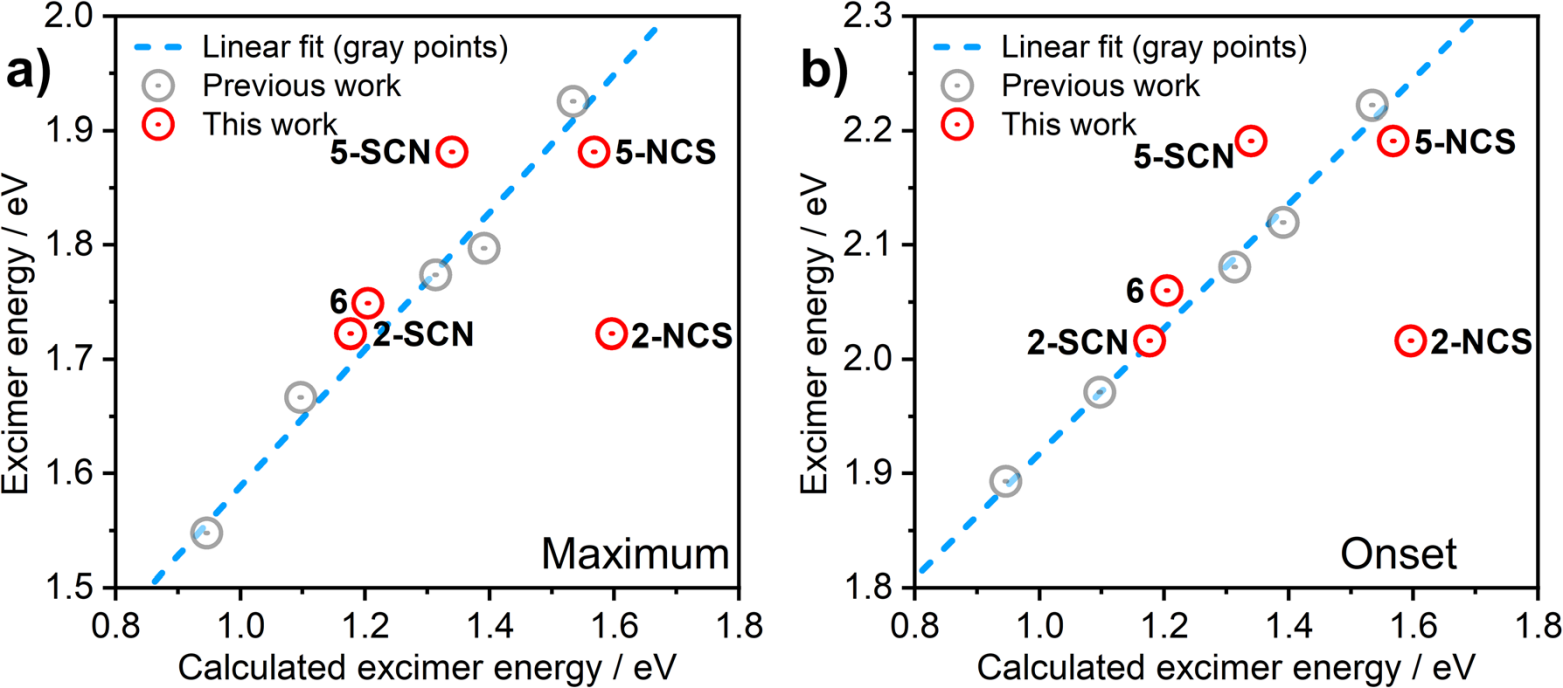
Correlation between the computed excimer energy and the experimental *λ*_max_ values (a) or emission onsets (b) obtained from the PL spectra in CH_2_Cl_2_ solutions. The red circles represent the data points relevant to the new complexes 2, 3, 5, and 6 presented in this work. The grey circles and the blue dashed line are data points and best linear fit, respectively, reproduced from our earlier work on related excimer-forming molecules of the same family.^36^

In order to understand the luminescence properties of excimers and aggregates formed from complexes 2, 3, 5, and 6, the triplet excited state of systems composed of increasing numbers of the constituent complexes arranged with a face-to-face orientation have been simulated using time-dependent density functional theory (TD-DFT). For 2 and 5, species comprising up to five molecular units (pentamers) have been studied; for 3 and 6, the largest species studied comprise two or three units respectively. The methodology described earlier is used,^36^ whereby the triplet excited state geometry of the aggregate is first obtained with TD-DFT using Orca^40–42^ software and BP86 (ref. 43)/def2-SVP,^44^ and the single-point energy calculation is then undertaken using B3LYP^45,46^/def2-SVP. An *anti* (head-to-tail) configuration of the neighbouring units is adopted for the model, as this revealed a rather better correlation with experiment than *syn* orientation in earlier studies. We use the previously established correlation between calculated excimer/dimer energy and experimental emission maximum or onset calibrated for CH_2_Cl_2_ solution to validate the calculations performed in this work (Fig. 6). The emissive state of all aggregates is presumed to be T_1_. A suffix NCS or SCN is used to distinguish between the calculated results for the N- and S-bound isomers of 2 and 5.

**Fig. 7:**
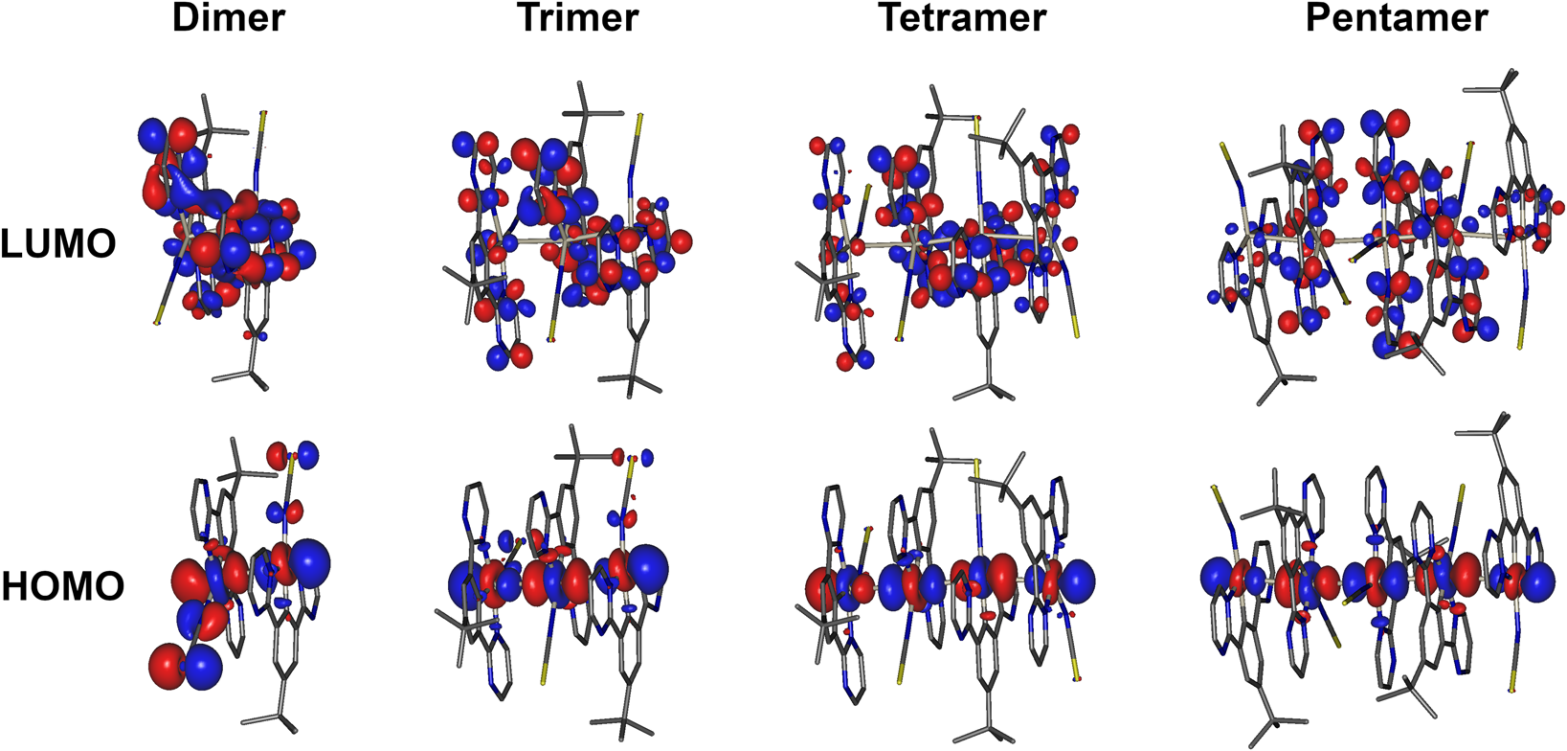
HOMO and LUMO iso surfaces in dimers, trimers, tetramers, and pentamers of complex 5(N-bound form) calculated at the T_1_ geometry.

HOMO and LUMO iso surfaces for aggregates of complex 5 comprising two, three, four or five molecules (*i.e.*, dimer, trimer, tetramer, pentamer) are presented in Fig. 7. Corresponding frontier MO plots for aggregates of other complexes and individual molecules are presented in the ESI (Fig. S4.1–S4.9†). The T_1_ and S_1_ states of all studied dimers/aggregates are characterised by a dominant contribution from the HOMO → LUMO transition (>95%), while other transitions have negligible coefficients. In all the dimers of the studied complexes, the HOMO is predominantly localised along the Pt–X (X = NCS/SCN or I) axis, while the LUMO is localised mostly on the cyclometallating ligand. This picture is broadly consistent with that found for isolated Pt(N^C^N)Cl complexes,^47^ but with lesser contribution to the HOMO from the metalated aryl ring. The contribution of the ancillary ligand to the HOMO is found to decrease as the size of the aggregate increases: the HOMO becomes dominated by the Pt d_*z*^2^_ orbitals from the tetramer upwards. Indeed, the most characteristic feature of the HOMO in face-to-face aggregates of planar Pt(II) complexes is the significant contribution from the d_*z*^2^_ orbital of the central atoms facing towards the opposite metal centre: a manifestation of Pt–Pt bimetallic interactions.^19,23,39^ Such configuration of HOMO and LUMO in the multimolecular excited states is a clear indication of the MMLCT character to the S_1_ and T_1_. The oscillator strength of the S_0_ → S_1_ transition increases with the number of aggregated molecules (Fig. S4.11 and Tables S4.1–4.7†), in agreement with the more pronounced MMLCT absorption bands in films of 2 and 5 forming larger aggregates with more red-shifted photoluminescence.

The predicted excimer energy is close to the experimental value for the iodo derivative 6 (Fig. 6). The aggregate energy calculated for complexes 2 and 5 generally depends on the binding mode of the ancillary ligand: the N-bound isomer yields a higher energy excited state than the S-bound. The reason for the difference in T_1_ energy between the two isomers is related to the different distribution of the HOMO. In the former, one of the –NCS groups bends at the nitrogen atom, leading to a lesser contribution of this co-ligand to HOMO, and so the extent of the molecular orbital is limited to two metal centres and one ancillary ligand. For the S-bound isomer, in contrast, the two ancillary ligands are equivalent and contribute to the HOMO. The difference in the calculated T_1_ energy between 2-SCN and 2-NCS exceeds 0.4 eV and, while the former demonstrates a perfect agreement with the experimental excimer PL spectrum, the latter does not match the experiment at all. This result strongly suggests that the binding of the ancillary ligand to the metal centre in complex 2 occurs through the sulphur atom, rather than the nitrogen, which agrees with the crystal structure presented in Fig. S3.1† showing the ancillary ligand to be S-bound. The predicted energies of the N and S-bound forms of 5 are much more similar than for 2, with only ∼0.1 eV energy difference between them.

**Fig. 8:**
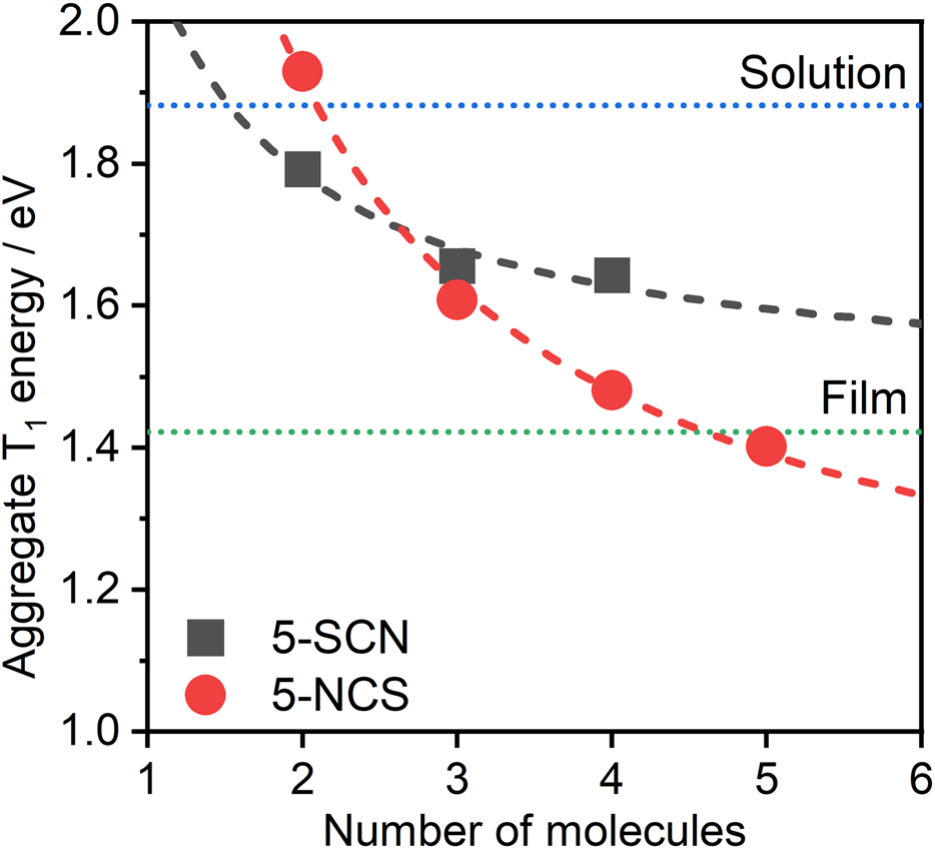
Theoretical PL maximum energy of multimolecular excited states of 5-NCS/5-SCN isomers as a function of the size of the aggregate. Note that we use the relationship from Fig. 6 for the prediction based on TD-DFT T_1_ energy of aggregates. Dotted lines represent the experimental energies of the excimer in CH_2_Cl_2_ solution (blue) and of the aggregate in neat film (green), estimated from the respective PL maxima. The determination of a reliable geometry of 5-SCN pentamer was not possible. Dashed lines indicate the best fit according to equation *E*(*n*) = *a* + *b*/*n*, where n is the number of aggregated molecules. The lines are provided simply as eye-guides for the trend.

The dependence of the calculated T_1_ energy on the size of the aggregate has also been probed using 5 as the model (Fig. 8). Comparison between the N- and S-bound isomeric aggregates of 5 reveals that, although the S-bound dimer displays the lower T_1_ energy, the N-bound T_1_ state falls in energy faster with the number of interacting units. For example, the PL from neat films of complex 5 could be readily explained as emanating from the T_1_ state of 5-NCS pentamers, while it would potentially require a significantly larger number of units to attain such a low T_1_ energy with the 5-SCN isomer. The trend in T_1_ energy *E*(*n*) as a function of *n* repeating units appears to roughly follow a function of the form *E*(*n*) = *a* + *b*/*n*, where *a* and *b* are constants (Fig. 8). From this we can estimate that the T_1_ energy at *n* → ∞ approaches ∼1.0 and ∼1.5 eV, respectively, for 5-NCS and 5-SCN, further suggesting that the 5-SCN aggregate is not a good model for 5 in film where *E* = 1.42 eV. Similar results for other complexes are presented in the ESI (Fig. S4.10†). In particular, N- and S-bound isomeric aggregates of four to six units of complex 2 are predicted to display similar PL maxima, despite the dimers showing a large difference in the T_1_ energy. In this case the experimental photoluminescence spectrum in film can be explained with aggregates of five to six molecules of the S-bound complex.

## Electroluminescence

**Fig. 9:**
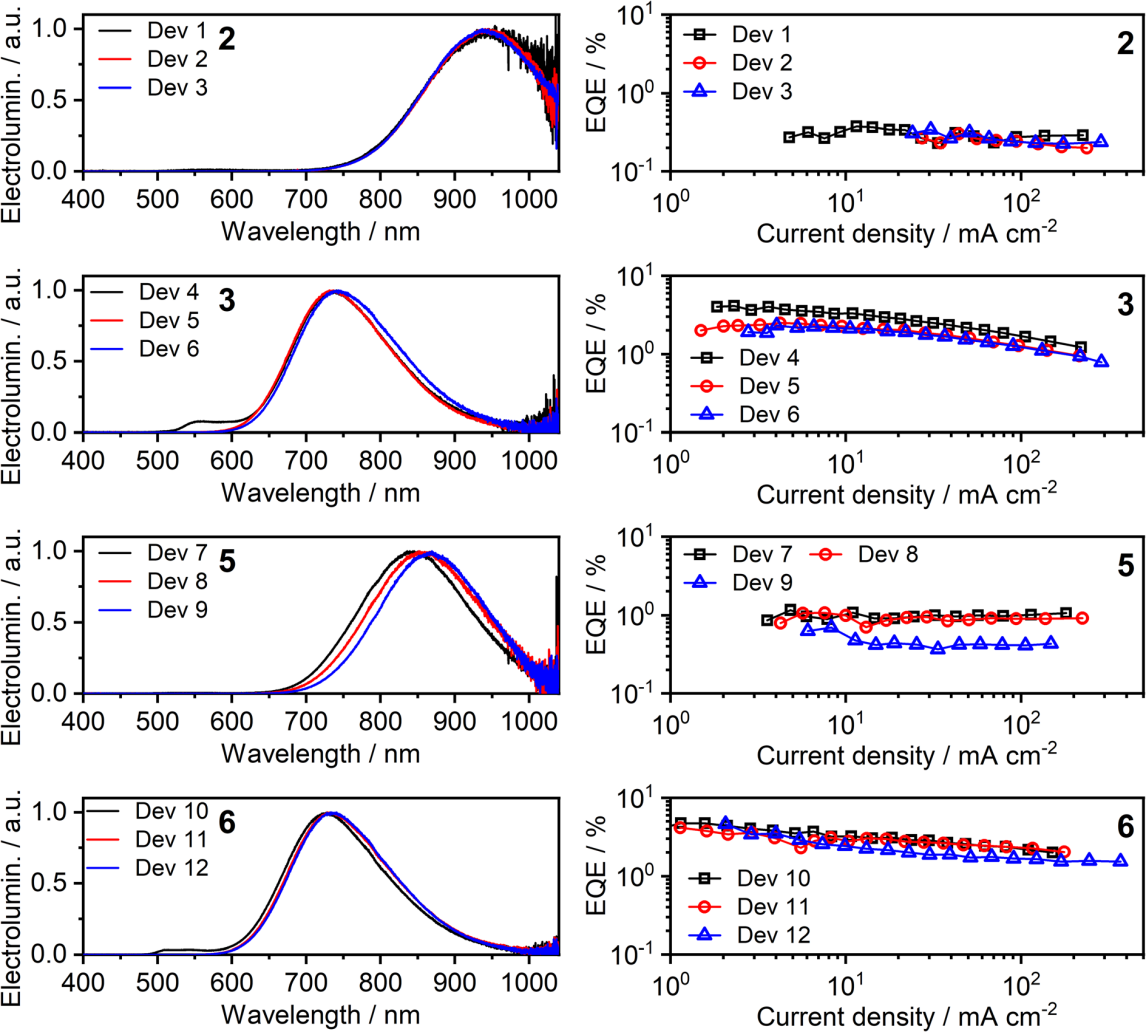
Electroluminescence spectra (left) and external quantum efficiency (EQE) (right) of Devices 1–12. The number in bold font identifies the complex used in the neat emissive layer.

All four of the investigated complexes (2, 3, 5, and 6) display strong NIR luminescence in films and thus were used as the emissive layer (EML) in OLEDs. In order to facilitate comparison with complexes 1 and 4 studied previously,^36^ we employ the optimised OLED architecture used earlier. In the structure ITO | HAT-CN (10 nm) | TSBPA (35 nm) | complex (*x* nm) | PO-T2T (50 nm) | LiF (0.8 nm) | Al (100 nm), “complex” identifies a neat layer of 2, 3, 5, or 6 as the emitting layer (EML), with a thickness *x* = 1, 2 or 10 nm. The structure confines charge-carrier recombination within the thin EML thanks to the hole-blocking PO-T2T and electron-blocking TSBPA. Electroluminescence spectra of the resulting twelve OLEDs, Devices 1–12, as well as external quantum efficiency (EQE) characteristics, are presented in Fig. 9; other characteristics are presented in the ESI, Fig. S7.1 and S7.2.† A summary of device performance is presented in Table 2.

**Table 2:** Characteristics of OLED devices fabricated with complexes 2, 3, 5, and 6 as emitters. Device structure: ITO|HAT-CN (10 nm)|TSBPA (35 nm)|complex (*x* nm)|PO-T2T (50 nm)|LiF (0.8 nm)|Al (100 nm)

Device	Complex	*Φ*_PL_ a	*x* b, nm	*λ*_EL_ c, nm	% *λ* > 700^d^ nm	EQE_max_^e^, %	Max. radiosity, mW cm^−2^
Dev 1	2	∼0.01^f^	1	945	>99	0.4	0.91
Dev 2			2	944	100	0.3	0.78
Dev 3			10	942	100	0.3	0.94
Dev 4	3	0.09 ± 0.02	1	558, 736	78	4.1	4.49
Dev 5			2	736	80	2.5	3.38
Dev 6			10	742	84	2.3	3.78
Dev 7	5	0.02 ± 0.02	1	842	98	1.1	2.88
Dev 8			2	857	99	1.1	3.04
Dev 9			10	868	100	0.7	2.54
Dev 10	6	0.12 ± 0.03	1	512, 541, 726	74	4.7	5.09
Dev 11			2	734	79	4.2	5.92
Dev 12			10	735	80	4.6	6.34

a Photoluminescence quantum yield of the emissive layer in nitrogen.

b Emissive layer (EML) thickness.

c Electroluminescence maxima.

d Percent of spectral power at wavelengths above 700 nm.

e Device maximum external quantum efficiency. Note the minimum error for EQE_max_ is ±0.1.

f The accuracy of the *Φ*_PL_ value is low in this instance due to the low intensity and long wavelength of the luminescence.

The OLED devices present remarkable NIR performance and they benefit from using a very thin layer of the platinum(ii) complex, leading to significant reduction in the consumption of the precious metal compound. Moreover, the device performance is generally seen to be similar or even better for *x* = 1 or 2 nm than for *x* = 10 nm. The maximum EQE of the devices closely follows the *Φ*_PL_ in film. Earlier reports of NIR-luminescent, face-to-face aggregated platinum(ii) complexes suggest that the molecules in film are oriented, leading to anisotropic luminescent behaviour of the emissive layer in OLED.^9,11^ In this case, the out-coupling ratio, typically 0.2–0.3 for non-oriented EML, increases to ∼0.3–0.4 as suggested in the literature.^11^ Our results are fully consistent with these earlier findings.

**Fig. 10:**
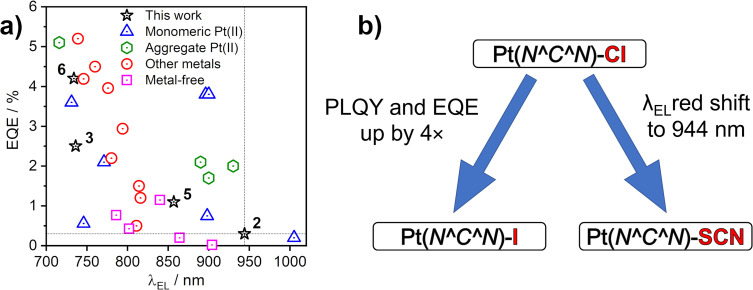
(a) Maximum external quantum efficiency (EQE) and electroluminescence spectrum maxima (*λ*_EL_) of sample NIR OLEDs from the literature that use platinum(ii) complexes or others as emitters indicated in the figure legend. See Table S7.1 in the ESI† for corresponding references. (b) Effects of the –X monodentate ligand on photoluminescence in film and electroluminescence.

The EL spectra are generally identical to the PL from the respective neat films. We observe a relatively small blue shift with thinner EML, or even a trace of monomolecular emission appearing, such as with complexes 3 and 6 featuring the ancillary iodide (500–600 nm band in Devices 4 and 10). Such variation of the electroluminescence spectrum with EML thickness suggests that as the layer becomes thinner, the probability that aggregates form is reduced (and they will be smaller ones if they do form), leading to luminescence from non-aggregated molecules being more likely to be observed. This behaviour is consistent with that reported recently by us for complexes 1 and 4.^36^ Device 4 featuring complex 3 in the EML displays an approximately four-fold increase in EQE with respect to its parent complex 1, despite a similar *λ*_EL_. We attribute that to the beneficial role of the heavier halogen in potentially suppressing molecular vibrations in the aggregates, hence slowing down non-radiative decay.^48,49^ Complexes 2 and 4 give the most red-shifted electroluminescence thanks to the formation of large aggregates featuring more than three molecular units, most likely four to six. Complex 5 gives a slightly longer wavelength EL *λ*_EL_ = 842–868 nm than the previously reported complex featuring the –NCS co-ligand,^35^ but retains a comparable EQE of roughly 1%. In the case of complex 2, an extremely long *λ*^max^_EL_ of 942 to 945 nm is observed in Devices 1–3, with an EQE ≈ 0.3–0.4%. This appears to be the longest wavelength EL reported for an OLED relying on a Pt(ii) complex aggregate, or indeed probably any other type of emitter, with an EQE > 0.2% (Fig. 10). The only example of a longer wavelength EL is a porphyrin derivative with *λ*_EL_ = 1005 nm, but the reported OLED EQE of 0.2% is lower.^16^ We believe that such long wavelength luminescence is owed to the fact that the electron-withdrawing peripheral –CF_3_ units stabilise the T_1_ energy in the multimolecular species, while the –NCS/SCN co-ligand promotes extension of the size of aggregates.

## Conclusions

We have presented four new excimer- and aggregate-forming platinum(ii) complexes of the Pt(N^C^N)X type and explored the effects of the co-ligand X on the properties of intermolecular excited states. Our study demonstrates that the ancillary ligand X (X = I or SCN/NCS) affects not only the energy of excimers in solution, but also the mode of aggregation in film. The use of X = I leads to a small red shift of the excimer spectrum with respect to X = Cl, but limits aggregation in the solid state to dimers, preventing larger aggregates from forming. The heavier iodide ligand reduces molecular vibrations leading to a pronounced increase in *Φ*_PL_ in film and OLED EQE with respect to films and devices employing the X = Cl analogues. We report on two different binding modes of the SCN ligand and demonstrate divergent behaviour of the isomeric N and S-bound excimers/aggregates through computational studies. The SCN/NCS co-ligand induces the formation of larger aggregates composed of at least four to six molecular units in film, leading to remarkably long wavelength luminescence > 850 nm. The diameter of the largest atom S (100 pm) in the thiocyanate ligand is smaller than that of I (140 pm), thus SCN may fit better in between the planes of the aggregated units, facilitating the formation of larger aggregates in film (Fig. S4.12†).

We use thin (1–10 nm) pristine emissive layers of our platinum(ii) complexes in proof-of-concept NIR OLED devices. The most efficient NIR OLED achieved has EQE = 4.7% and reaches maximum radiosity of 5.09 mW cm^−2^ with *λ*^max^_EL_ = 726 nm. But, most remarkably, we demonstrate exceptionally long-wavelength EL for an OLED device using an aggregating Pt(ii) emitter, with *λ*^max^_EL_ = 944 nm and maximum EQE = 0.3 ± 0.1%.

## Data availability

Our supporting research data is available from the Durham Research Online DATAsets Archive (DRO-DATA) open data repository. DOI: 10.15128/r202870v95d.

## Author contributions

R. J. S. – investigation (synthesis, photophysics), writing – original draft, writing – review & editing; P. P. – conceptualization, formal analysis, investigation (photophysics, calculations, OLED devices), visualization, writing – original draft, writing – review & editing; D. Y. – investigation (X-ray diffraction); F. B. D. – Funding acquisition, project administration, resources, validation, writing – original draft, writing – review & editing; J. A. G. W. – conceptualization, funding acquisition, project administration, supervision, writing – original draft, writing – review & editing.

## Conflicts of interest

The authors have no conflicts to declare.

## Supplementary Material

SC-013-D2SC05023D-s001

SC-013-D2SC05023D-s002

SC-013-D2SC05023D-s003

SC-013-D2SC05023D-s004

SC-013-D2SC05023D-s005

SC-013-D2SC05023D-s006

SC-013-D2SC05023D-s007

SC-013-D2SC05023D-s008

SC-013-D2SC05023D-s009

SC-013-D2SC05023D-s010

SC-013-D2SC05023D-s011

SC-013-D2SC05023D-s012

SC-013-D2SC05023D-s013

SC-013-D2SC05023D-s014

SC-013-D2SC05023D-s015

SC-013-D2SC05023D-s016

SC-013-D2SC05023D-s017

SC-013-D2SC05023D-s018

SC-013-D2SC05023D-s019

SC-013-D2SC05023D-s020

SC-013-D2SC05023D-s021

SC-013-D2SC05023D-s022

SC-013-D2SC05023D-s023

SC-013-D2SC05023D-s024

SC-013-D2SC05023D-s025
