# Supplementary material for: Near-infrared electroluminescence beyond 940 nm in Pt(N^C^N)X complexes: influencing aggregation with the ancillary ligand X
Source: Chem Sci. 2022 Nov 11;13(45):13600–10. doi: 10.1039/d2sc05023d (PMC9682897; doi:10.1039/d2sc05023d)
Supplement: SC-013-D2SC05023D-s024 [file SC-013-D2SC05023D-s024.pdf]

Electronic Supplementary Material (ESI) for Chemical Science.
This journal is © The Royal Society of Chemistry 2022

Supporting information

Near-Infrared Electroluminescence beyond 940 nm in Pt(*N^C^N*)X Complexes: Influencing Aggregation with the Ancillary Ligand X

Rebecca J. Salthouse, Piotr Pander, Dmitry S. Yufit, Fernando B. Dias, and J. A. Gareth Williams

Table of contents

1. General 2
2. Synthesis 5
3. Crystallography 12
4. Computations 14
5. Photophysics 24
   a) **Solution state** 24
   b) **Solid film** 25
6. Electrochemistry 35
7. OLED devices 36
8. Influence of ancillary ligand - summary 39
9. References 40

1. General

Theory

To assist the interpretation of the experimental results, we have performed density functional theory (DFT) and time-dependent density functional theory (TDDFT) simulations with Tamm-Dancoff approximation (TDA) on the monoplatinum(II) complexes using the ORCA 4.2.1 quantum chemistry software.[1–3] All molecular orbital (MO) iso surfaces were visualised using Gabedit 2.5.0[4] or Avogadro 1.2.0.[5,6]

Geometry optimisations of complexes **2**, **3**, **5** and **6** in ground state were performed at the B3LYP[7,8]/def2-SVP[9] level of theory. Triplet excited state (T_1) geometries of aggregates were performed at the BP86[10]/def2-SVP[9] level of theory using the RI keyword, and def2-SVP/C[11] and def2/J[12] auxiliary basis sets. Single point energy (SPE) calculations were performed at the B3LYP[7,8]/def2-SVP[9] level of theory with the aid of RIJCOSX[13,14] approximation and using CPCM for CH_2Cl_2 in all cases. All calculations were performed using a dense grid (Grid6, GridX6), and *very tight* geometry and SCF convergence criteria and using the atom-pairwise dispersion correction with the Becke-Johnson damping scheme (D3BJ).[15,16] Frequency calculations found all respective optimised geometries to be local minima.

Electrochemistry

Cyclic voltammetry was conducted in a three-electrode, one-compartment cell. All measurements were performed using 0.1 M Bu_4NBF_4 (99%, Sigma Aldrich, dried) solution in dichloromethane (ExtraDry AcroSeal®, Acros Organics). All solutions were bubbled with nitrogen prior to measurement and the measurement was conducted in nitrogen atmosphere. Electrodes: working (Pt disc d = 1 mm), counter (Pt wire), reference (Ag/AgCl calibrated against ferrocene). All cyclic voltammetry measurements were performed at room temperature with a scan rate of 50 mV s^{-1}.

The ionization potential (IP) and electron affinity (EA) are obtained from onset redox potentials; these figures correspond to HOMO and LUMO values, respectively. The ionization potential is calculated from onset oxidation potential $IP = E_{ox}^{CV} + 5.1$ [eV] and the electron affinity is calculated from onset reduction potential $EA = E_{red}^{CV} + 5.1$ [eV].[17–20] We assume an uncertainty of ±0.02 V.

Photophysics

Photophysical measurements in solution. Absorption spectra in solution were recorded with a double-beam Biotek Instruments XS spectrometer using quartz cuvettes of 1 cm pathlength. Emission spectra in solution were recorded in 1 cm pathlength quartz cuvettes modified for connection to a vacuum line.

Solutions were degassed by a minimum of three freeze-pump-thaw cycles to a pressure of < 10^{-2} mbar at 77 K, as measured by a Pirani gauge. The spectra were measured using a Jobin Yvon Fluromax-2 spectrometer, equipped with a Hamamatsu R928 photomultiplier tube (PMT). The emission spectra shown and the accompanying λ_{max} data were obtained after correction for the wavelength-dependence of the emission monochromator and detection system. Luminescence lifetimes were obtained upon excitation with a laser diode at 405 nm. The emitted light was detected at right angles to the excitation beam, after passage through a monochromator, using a Peltier-cooled R928 PMT operating in single-photon counting mode.

Photophysical measurements in film. Thin films for photophysical studies were obtained by thermal evaporation on sapphire substrates. Absorption spectra in film were recorded with UV-3600 double beam spectrophotometer (Shimadzu). Photoluminescence (PL) spectra in films were recorded using a QePro compact spectrometer (Ocean Optics) in nitrogen flow. Time-resolved decays in solution and film were recorded with a Horiba DeltaFlex TCSPC system using a 405 nm DeltaDiode light source. Temperature-dependent experiments were conducted using a liquid nitrogen cryostat VNF-100 (sample in flowing vapour, Janis Research) under nitrogen atmosphere, while measurements at room temperature were recorded under vacuum in the same cryostat. Photoluminescence quantum yield in film was obtained using an integrating sphere (Labsphere) coupled with a 365 nm LED light source and a QePro (Ocean Optics) detector.

OLED devices

OLEDs were fabricated by vacuum thermal evaporation method. The hole injection layer (HAT-CN), was deposited first, while hole transport/electron blocking layer (TSBPA) followed. Subsequently, respective platinum complexes were evaporated as the pristine emissive layer (EML). PO-T2T served as the electron transport/hole blocking layer, while LiF was the electron injection layer and Al the cathode. Devices with 4 × 2 mm pixel size were fabricated. Dipyrazino[2,3-f :2',3'-h]quinoxaline-2,3,6,7,10,11-hexacarbonitrile (HAT-CN, Lumtec, sublimed), 4,4'-(diphenylsilanediyl)bis(*N*,*N*-diphenylaniline) (TSBPA, Lumtec, sublimed), 2,4,6-tris[3-(diphenylphosphinyl)phenyl]-1,3,5-triazine (PO-T2T, LUMTEC), 1,3-bis(carbazol-9-yl)benzene (mCP, Lumtec, sublimed), 1,3,5-tri[(3-pyridyl)-phen-3-yl]benzene (TmPyPB, Lumtec), LiF (99.995%, Sigma Aldrich), and Aluminium pellets (99.9995%, Lesker) were purchased from the companies indicated in parentheses. We used pre-patterned indium-tin-oxide (ITO) coated glass substrates with a sheet resistance of 20 Ω cm^{-2} and ITO thickness of 100 nm. The substrates were pre-cleaned with oxygen plasma before use. All organic and inorganic layers were thermally evaporated using Kurt J. Lesker Spectros II deposition system at 10^{-6} mbar base pressure. All organic materials and aluminium were deposited at a rate of 1 Å s^{-1} except for

the EML which was deposited at a rate of 0.1-0.3 1 Å s^{-1}. The LiF layer was deposited at a rate of 0.1–0.2 Å s^{-1}. Characterisation of OLED devices was conducted in a 10 inch integrating sphere (Labsphere) connected to a Source Measure Unit Keithley 2400 and coupled with a spectrometer USB4000 (Ocean Optics). Further details are available in reference [21].

Materials and methods for Synthesis

Commercial chemicals were used as supplied without further purification. Reactions requiring an inert atmosphere were carried out using Schlenk-line techniques under an atmosphere of argon or nitrogen gas. Thin-layer chromatography analysis was performed on F_{254} silica plates and visualised by UV irradiation at 254 and 365 nm.

NMR spectra were recorded on a Bruker Avance-400 spectrometer (400 MHz), a Varian VNMRS-600 (600 MHz) or a Varian VNMRS-700 (700 MHz). Two-dimensional NMR experiments, including homonuclear correlation spectroscopy (COSY), heteronuclear multiple bond correlation spectroscopy (HMBC) and heteronuclear single quantum coherence spectroscopy (HSQC), were used to aid assignments. Chemical shift values (δ) are reported in parts per million (ppm) and coupling constants (J) in Hz. C^q denotes a quaternary carbon and the spectra are referenced to residual solvent peaks: $CDCl_3$ = 7.26 ppm (^{1}H), 77.16 ppm (^{13}C); DMSO = 2.50 ppm (^{1}H), 39.52 ppm (^{13}C). Multiplicity is as follows: s (singlet); d (doublet); t (triplet); q (quartet) and m (multiplet).

Electrospray ionisation mass spectra (ESI) were recorded using a Waters Acquity® TQD Tandem Quadrupole mass spectrometer with column using water containing formic acid (0.1% /v) and either acetonitrile or methanol as the carrier solvent. Atmospheric pressure solids analysis probe (ASAP) ionisation mass spectra were obtained using an LCT Premier XE mass spectrometer and an Acquity® UPLC from Waters Ltd at 350°C. High-resolution mass spectra were obtained using a Quantum time-of-flight (QTof) mass spectrometer.

2. Synthesis

The parent chloro complexes **1** and **4** were prepared as reported in our recent work that included a study of these systems.[22] They were used as the starting materials to obtain the new complexes reported in the present work: **2** and **3** from **1**, and **5** and **6** from **2**. The appropriate chloro-complex **1** or **2** was suspended in acetone; silver triflate (AgOTf) was added and the mixture stirred at ambient temperature for 90 minutes. The silver chloride that precipitated was removed from the solution by centrifugation. The potassium salt of the desired ancillary ligand (KI or KSCN) was added to the acetone solution and stirred at ambient temperature for 2 h. The product complex precipitated from the reaction and was isolated by centrifugation, and then washed with water, ethanol and diethyl ether. The solid was extracted into CH_2Cl_2, the resulting solution centrifuged, and the solvent was finally removed under reduced pressure to give the desired product.

Complex 2

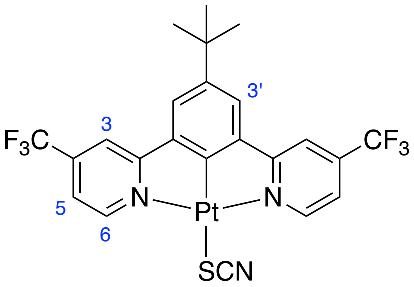

This complex was obtained from **1** (50 mg, 0.076 mmol), AgOTf (24 mg, 0.092 mmol) and KSCN (15 mg, 0.150 mmol) to give the product as a green yellow solid (33 mg, 64%); δ H (400 MHz, CD_2Cl_2) 8.99 (2H, d, J 5.7, H^6), 7.99 (2H, s, H^3), 7.69 (2H, s, $H^{3'}$), 7.59 (2H, d, J 5.7, H^5), 1.48 (9H, s, $H^{t\text{-Bu}}$); δ F (376 MHz, CD_2Cl_2) -65.46 (s); δ C (151 MHz, HSQC, CD_2Cl_2) 153.2 (C^6), 122.9 ($C^{3'}$), 119.7 (C^5), 115.7 (C^3), 30.9 (CH_3); the solubility was too poor to resolve the quaternary carbons; HRMS (AP^+) *m/z* 676.0767 $[M+H]^+$, calc. for 676.0752 $[C_{23}H_{18}N_3F_6S^{194}Pt]$; ν_{max} 2074 cm^{-1} (SCN str); Anal. calcd for $C_{23}H_{17}F_6N_3PtS{\cdot}0.1CH_2Cl_2$: C, 40.50; H, 2.53; N, 6.13 %; found C, 40.35; H, 2.46; N, 6.08 %.

Complex 3

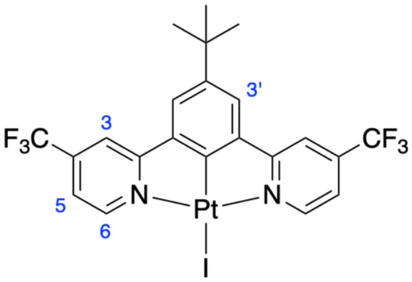

This complex was obtained from **1** (50 mg, 0.076 mmol), AgOTf (24 mg, 0.092 mmol) and KI (38 mg, 0.229 mmol) to give the product as a yellow solid (41 mg, 60%); δ H (600 MHz, CD_2Cl_2) 9.90 (2 H, dd, J 5.8, H^6), 8.90 (2 H, dd, J 4.8, 2.3, H^4), 7.97 (2 H, s, $H^{3'}$), 7.26 (2 H, dd, J 5.8, 4.8, H^5), 1.45 (9 H, s, $H^{t\text{-}Bu}$); δ F (376 MHz, $CDCl_3$) -65.50 (s); δ C (151 MHz, HSQC, CD_2Cl_2) 158.3 (C^6), 123.2 ($C^{3'}$), 119.9 (C^5), 115.3 (C^3), 30.9 (CH_3); the solubility was too poor to resolve the quaternary carbons; MS ASAP (AS^+) *m/z* 659.1 ($[M\text{-}I+MeCN]^+$, 100%); HRMS (AS^+) *m/z* 658.1196 $[M+H]^+$, calc. for 658.1188 $[C_{24}H_{20}N_3F_6{}^{194}Pt]$; Anal. calcd for $C_{22}H_{17}F_6IN_2Pt$: C, 35.45; H, 2.30; N, 3.76 %; found C, 35.51; H, 2.29; N, 3.65 %.

Complex 5

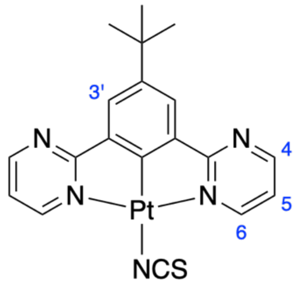

This complex was obtained from **4** (25 mg, 0.048 mmol), AgOTf (15 mg, 0.060 mmol) and KSCN (47 mg, 0.480 mmol) to give the product as a burgundy red/purple solid (11 mg, 42%); δ H (600 MHz, CD_2Cl_2) 8.96 (2 H, dd, J 4.8, 2.3, H^4), 8.89 (2 H, dd, J 5.7, 2.3, H^6), 7.87 (2 H, s, $H^{3'}$), 7.34 (2 H, dd, J 5.6, 4.8, H^5), 1.43 (9H, s, *t*-Bu); δ C (151 MHz, HSQC, CD_2Cl_2) 159.4 (C^4), 158.0 (C^6), 126.8 ($C^{3'}$), 119.3 (C^5), 30. (CH_3); the solubility was too poor to resolve the quaternary carbons; MS ASAP (AP^+) *m/z* 525.1 $[M\text{-}NCS+MeCN]^+$; HRMS (AP^+) *m/z* 524.1336 $[M\text{-}NCS+MeCN]^+$, calc. for 524.1345 $[C_{20}H_{20}N_5{}^{194}Pt]$; ν_{max} 2108 cm^{-1} (NCS str.); Anal. calcd for $C_{19}H_{17}N_5PtS{\cdot}0.1CH_2Cl_2$: C, 41.63; H, 3.15; N, 12.71 %; found C, 41.63; H, 3.10; N, 12.72 %.

Complex 6

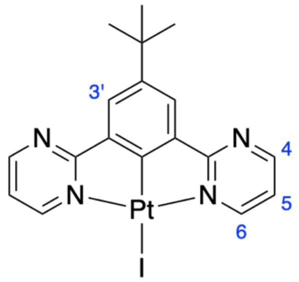

This complex was prepared from **4** (25 mg, 0.048 mmol), AgOTf (15 mg, 0.060 mmol) and KI (80 mg, 0.480 mmol) to give the product as a dark green solid (8 mg, 27%); δ H (600 MHz, CD_2Cl_2) 9.90 (2 H,

dd, J 5.8, H^6), 8.90 (2 H, dd, J 4.8, 2.3, H^4), 7.97 (2 H, s, $H^{3'}$), 7.26 (2 H, dd, J 5.8, 4.8, H^5), 1.45 (9 H, s, $H^{t\text{-Bu}}$); δ C (151 MHz, HSQC, CD_2Cl_2) 163.2 (C^6), 158.2 (C^4), 126.9 ($C^{3'}$), 119.3 (C^5), 31.0 (CH_3); the solubility was too poor to resolve the quaternary carbons; MS ASAP (AP^+) *m/z* 525.1 ($[M\text{-}I+MeCN]^+$, 100%), 612.0 ($[M+H]^+$, 2%); HRMS (AP^+) *m/z* 524.1349 $[M\text{-}I+MeCN]^+$, calc. for 524.1345 [$C_{20}H_{20}N_5{}^{194}Pt$]; Anal. calcd for $C_{19}H_{17}N_5PtS{\cdot}0.2CH_2Cl_2$: C, 34.79; H, 2.79; N, 8.92 %; found C, 34.90; H, 2.64; N, 8.70 %.

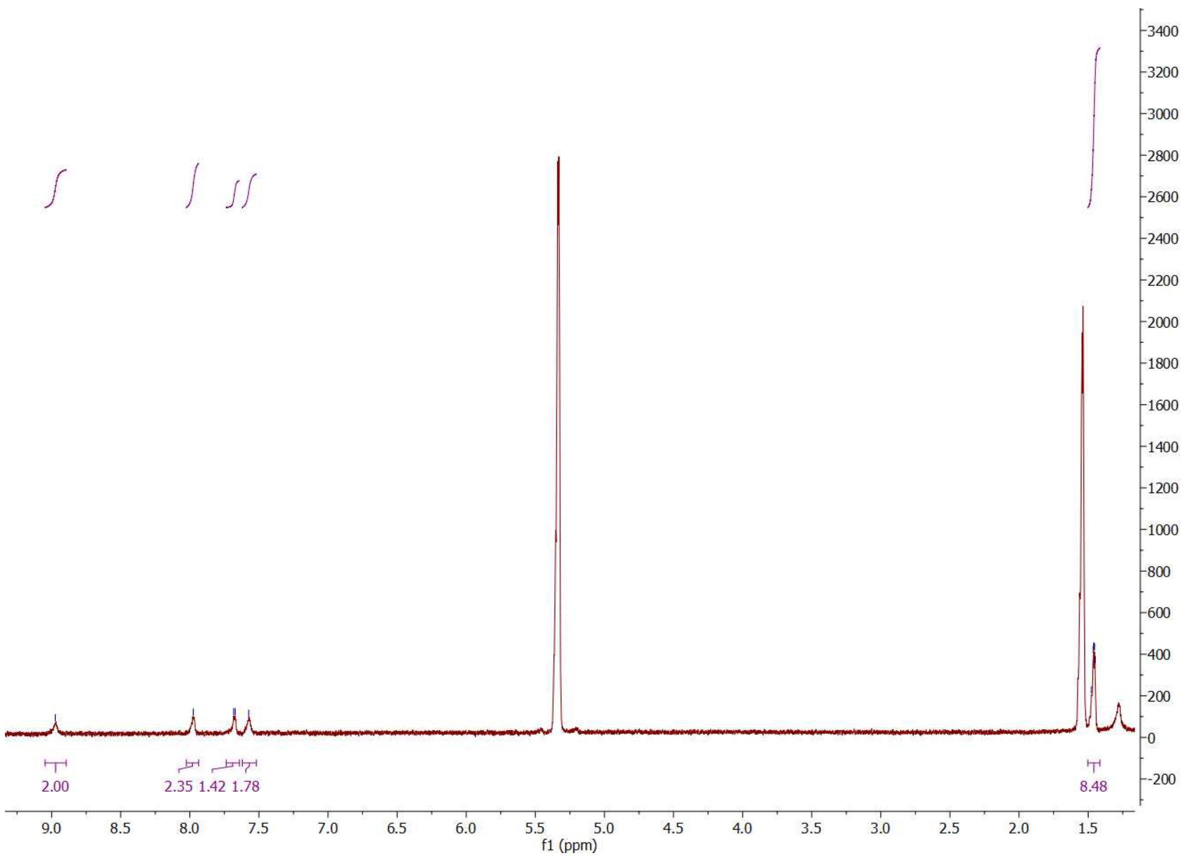

Figure S2.1. ^{1}H NMR spectrum of Complex **2** in CD_2Cl_2 at 700 MHz.

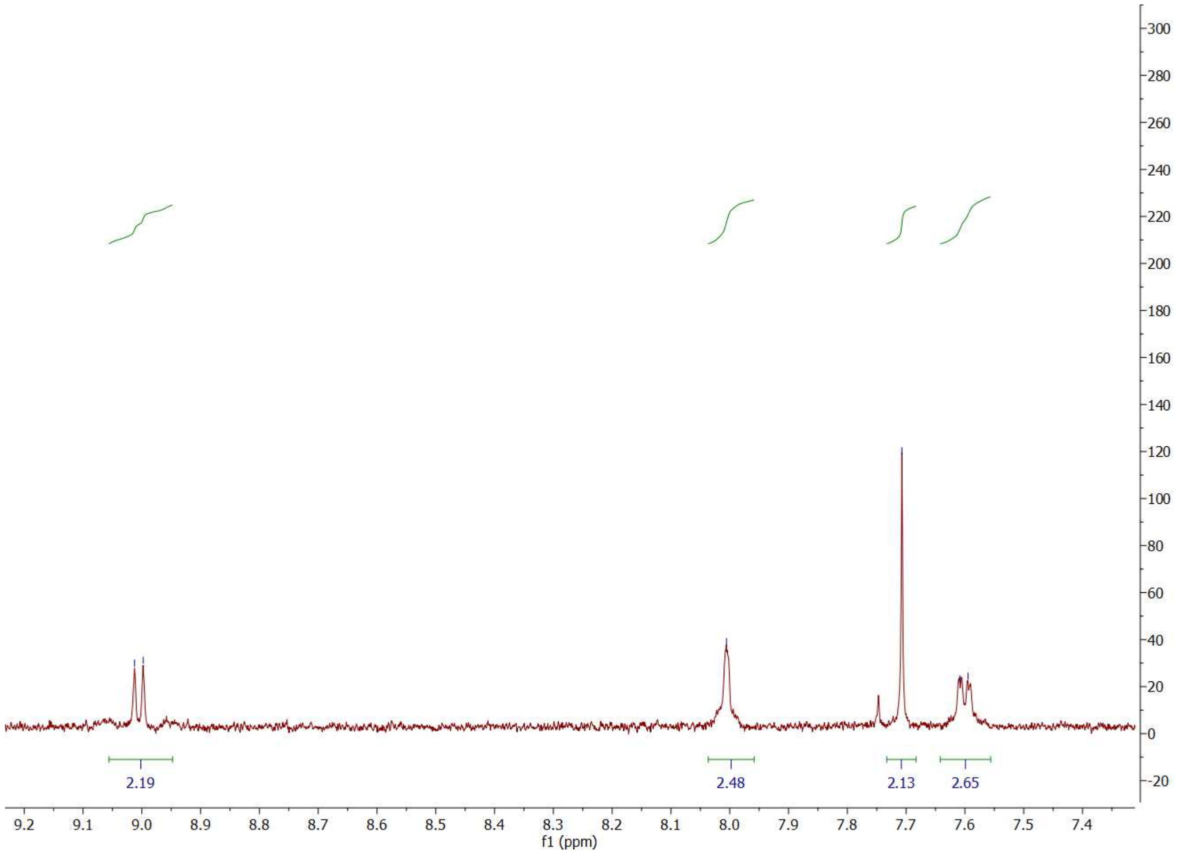

Figure S2.2. ^{1}H NMR spectrum of Complex **2** in CD_2Cl_2 at 400 MHz.

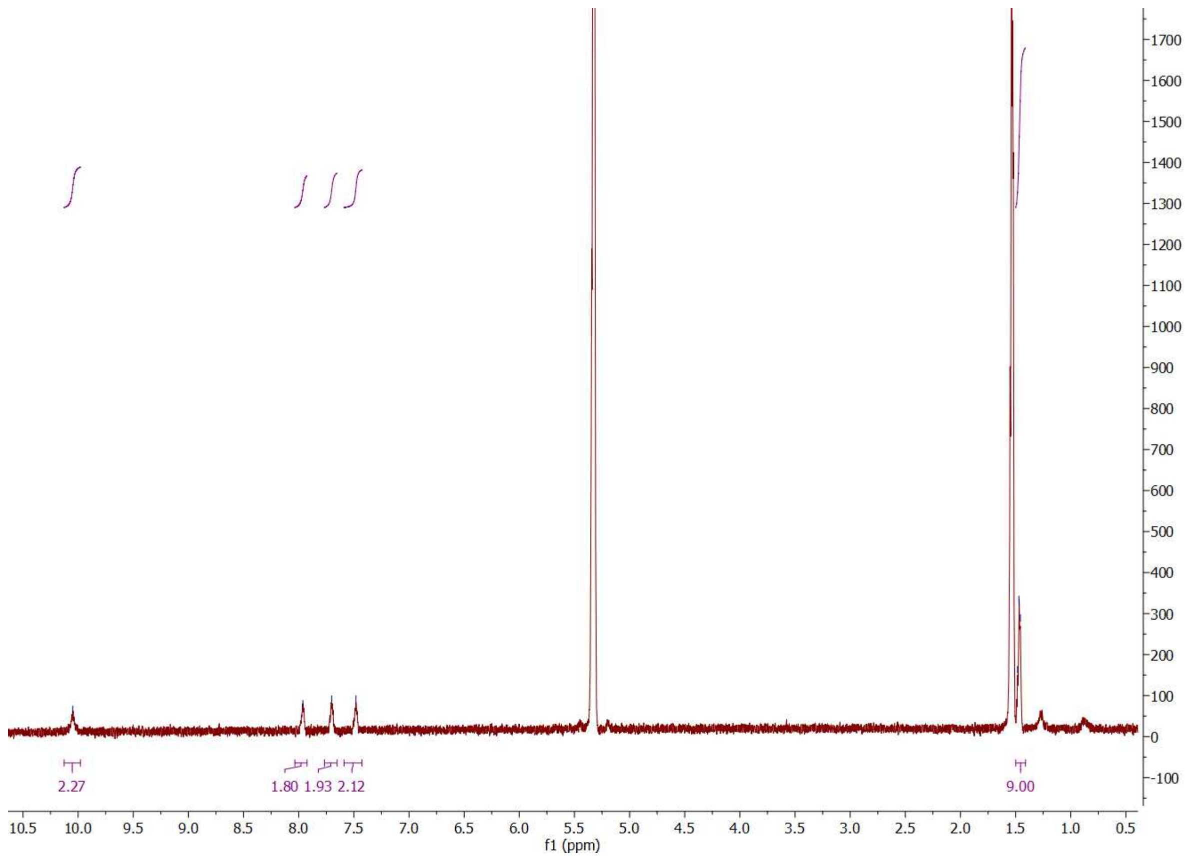

Figure S2.3. ^{1}H NMR spectrum of Complex **3** in CD_2Cl_2 at 700 MHz.

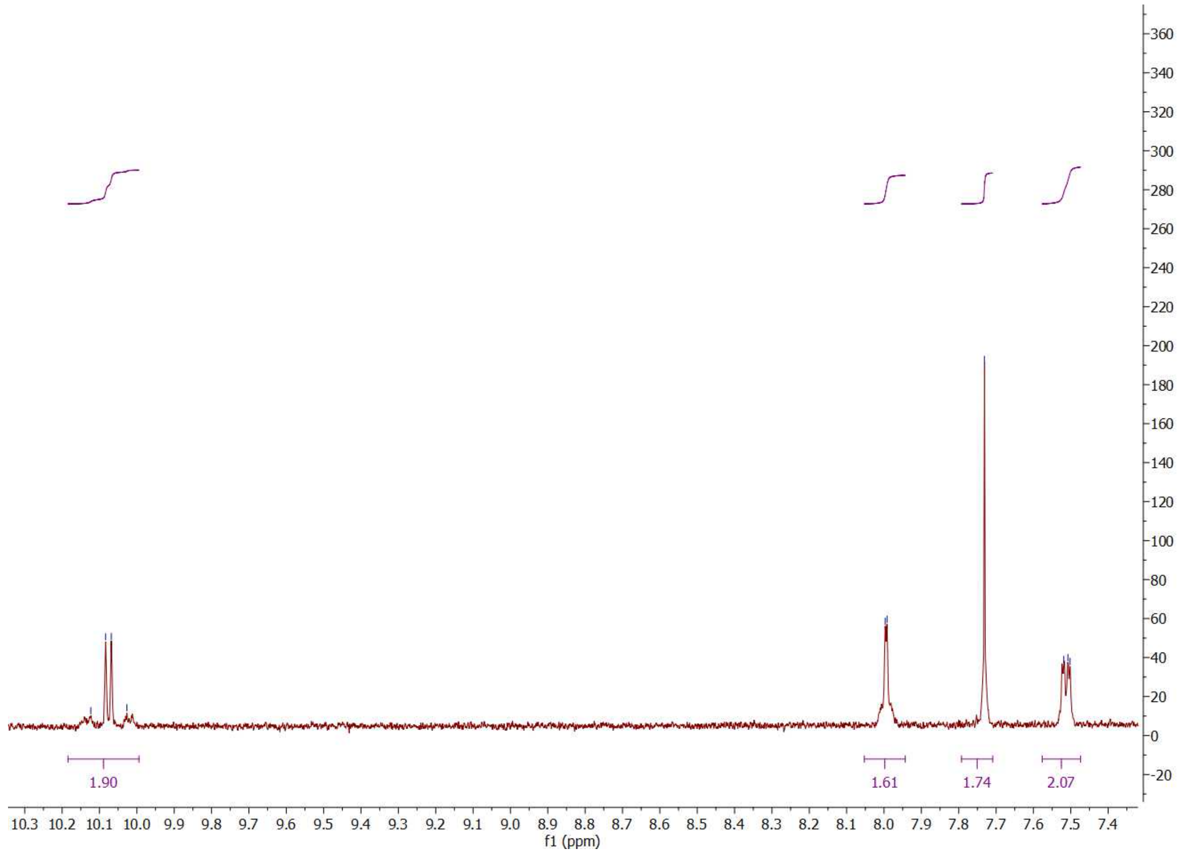

Figure S2.4. ^{1}H NMR spectrum of Complex **3** in CD_2Cl_2 at 400 MHz.

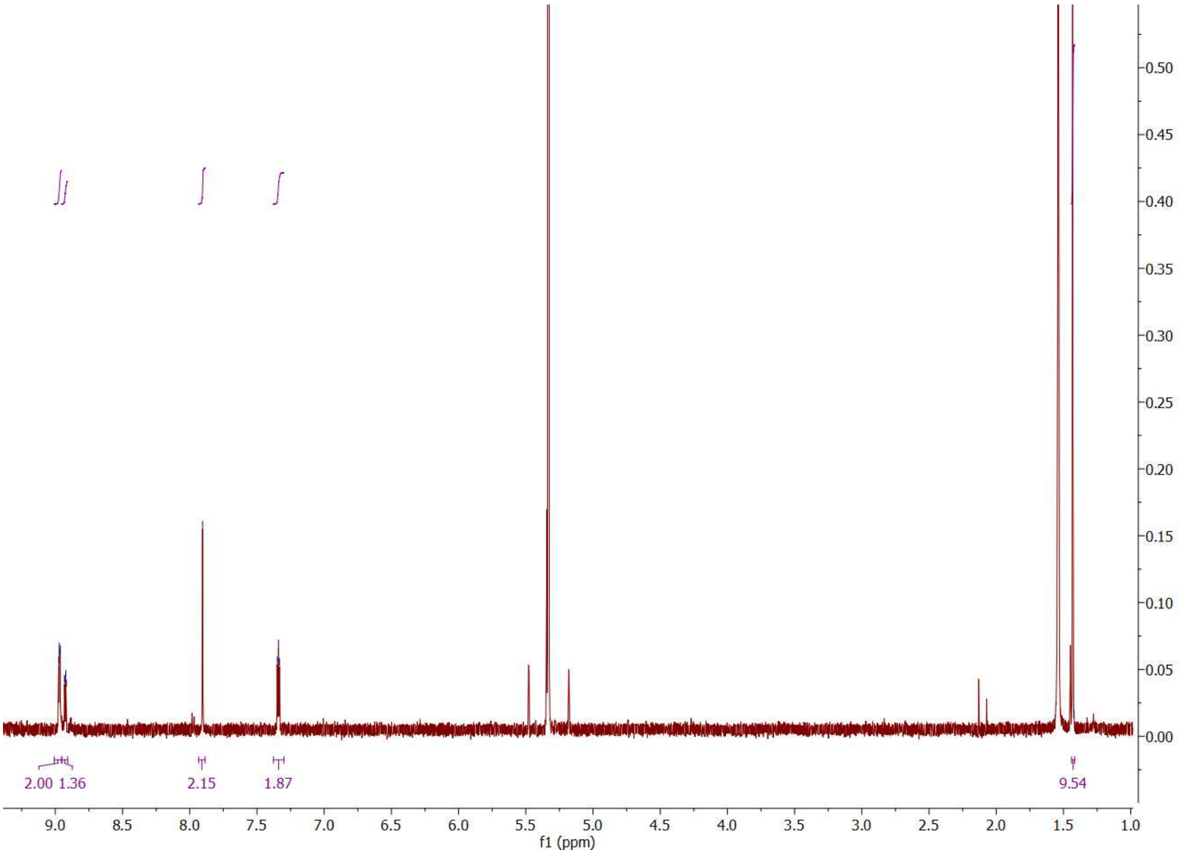

Figure S2.5. ^{1}H NMR spectrum of Complex **5** in CD_2Cl_2 at 600 MHz.

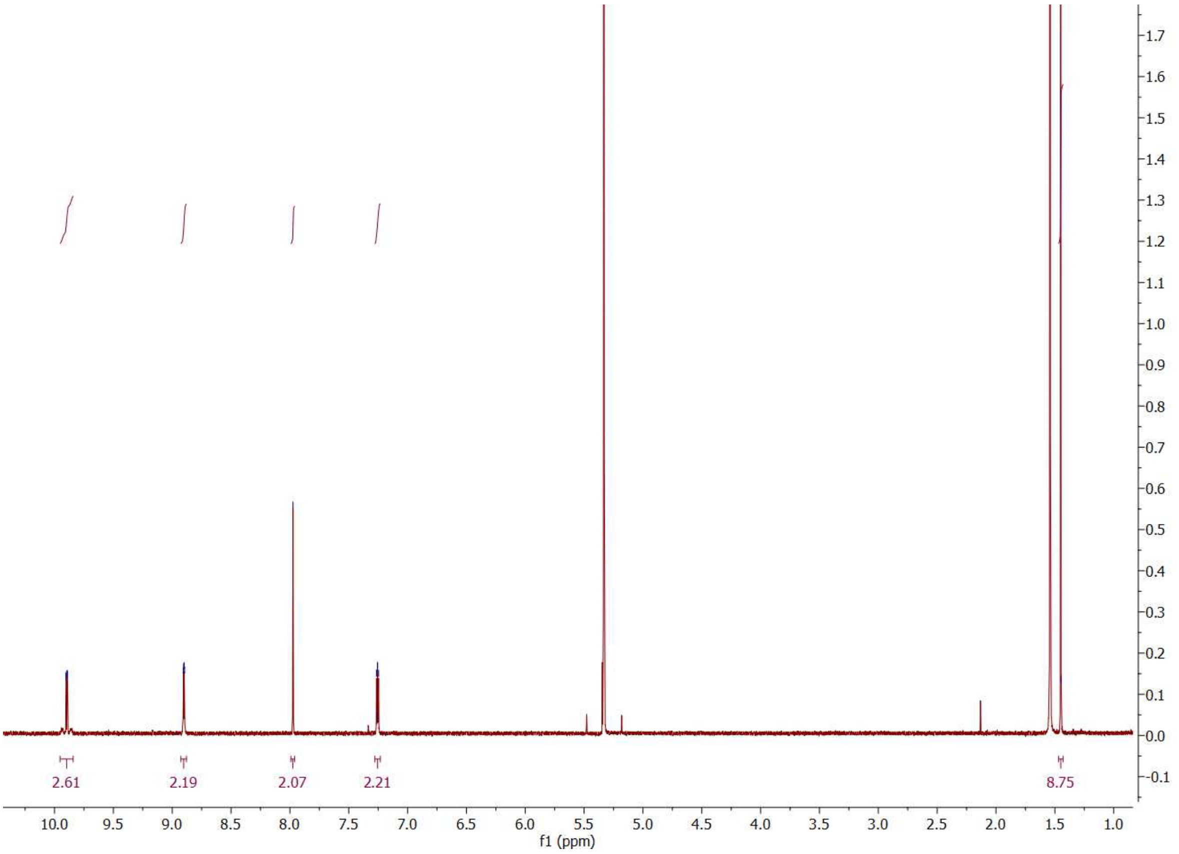

Figure S2.6. ^{1}H NMR spectrum of Complex **6** in CD_2Cl_2 at 600 MHz.

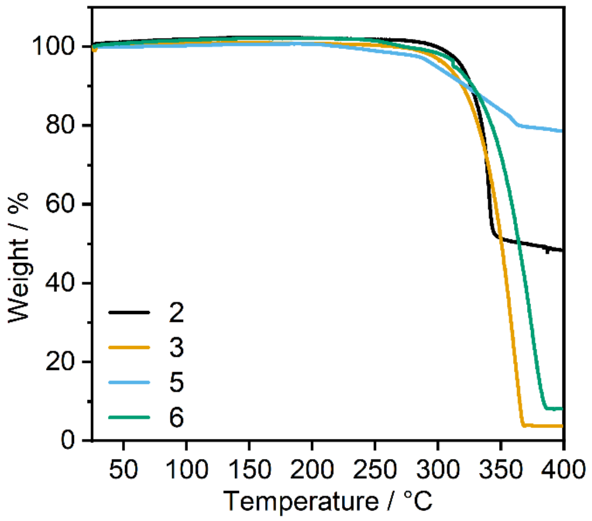

Figure S2.7. Thermogravimetric (TGA) curves recorded for complexes **2**, **3**, **5**, and **6**.

3. Crystallography

The X-ray single crystal data were collected using λMoKα radiation (λ =0.71073Å) on a Bruker D8Venture (Photon III MM C14 CPAD detector, IμS-III-microsource, focusing mirrors) 4-circle diffractometer equipped with a Cryostream (Oxford Cryosystems) open-flow nitrogen cryostat at a temperature of 120.0(2)K. Both structures were solved by direct methods and refined by full-matrix least squares on F^2 for all data using Olex2[23] and SHELXTL[24] software. All non-hydrogen atoms were refined in anisotropic approximation, the hydrogen atoms were placed in calculated positions and refined in riding mode. The atoms of the disordered *t*Bu group in the structure of **2** were refined isotropically with fixed site occupational factors (SOFs) (0.6:0.4). Same fixed SOFs were applied for atoms of Cl and SCN disordered group respectively, but these atoms were refined in restrained (ISOR) anisotropic mode. Crystal data and parameters of refinement are listed in Table S3.1. Crystallographic data for the structures have been deposited with the Cambridge Crystallographic Data Centre as supplementary publication CCDC-2204977, 2204978.

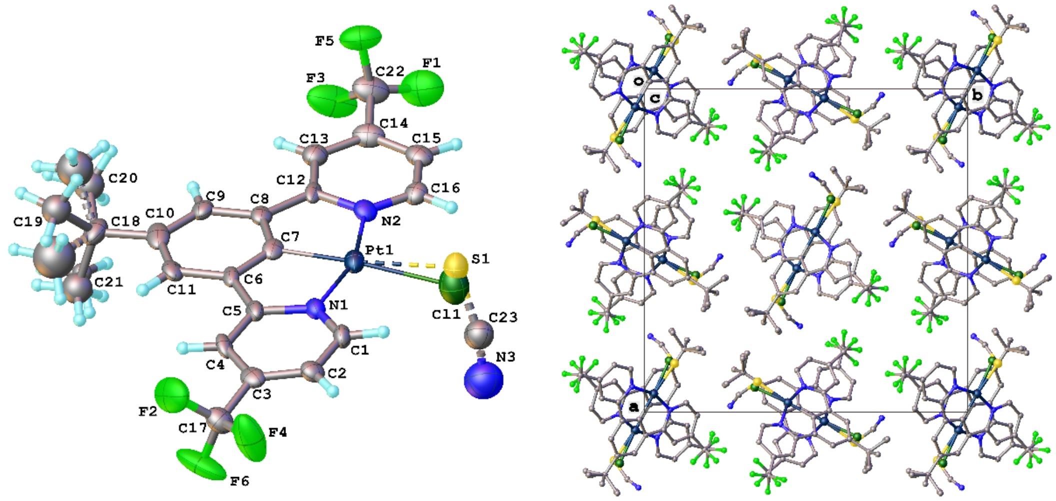

Figure S3.1. Molecular and crystal structure of complex **2**.

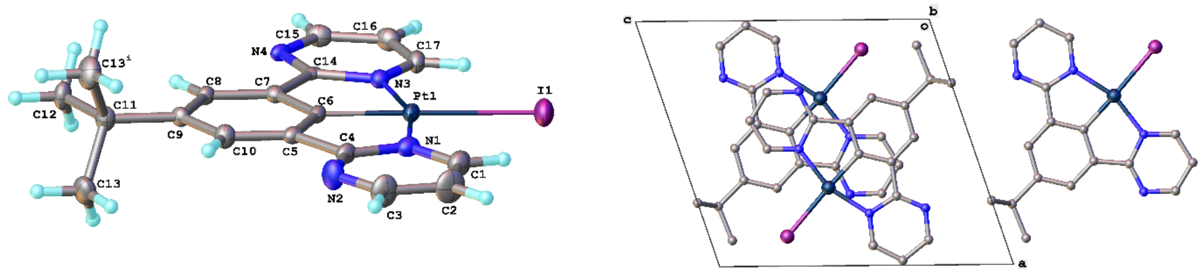

Figure S3.2. Molecular and crystal structure of complex **6**.

Table S3.1. Crystal data and structure refinement for complexes **2** and **6.**

	**Complex 2** (co-crystallised with complex **1**)	**Complex 6**
Identification code	21srv377	21srv399
Empirical formula	$C_{22.4}H_{17}Cl_{0.6}F_6N_{2.4}PtS_{0.4}$	$C_{18}H_{17}IN_4Pt$
Formula weight	662.97	611.34
Temperature/K	120.0	120.0
Crystal system	tetragonal	monoclinic
Space group	$P4_2/n$	$P2_1/m$
a/Å	21.2092(6)	10.8783(3)
b/Å	21.2092(6)	6.7723(2)
c/Å	9.3816(3)	12.3368(3)
α/°	90	90
β/°	90	108.5190(10)
γ/°	90	90
Volume/Å^3	4220.1(3)	861.80(4)
Z	8	2
ρ_{calc}g/cm^3	2.087	2.356
μ/mm^{-1}	6.833	9.939
F(000)	2534.0	568.0
Crystal size/mm^3	0.23 × 0.02 × 0.01	0.09 × 0.06 × 0.005
Radiation	Mo Kα (λ = 0.71073)	Mo Kα (λ = 0.71073)
2Θ range for data collection/°	3.84 to 54.998	3.948 to 59.998
Index ranges	$-27 \leq h \leq 27, -27 \leq k \leq 27, -12 \leq l \leq 12$	$-15 \leq h \leq 15, -9 \leq k \leq 9, -17 \leq l \leq 17$
Reflections collected	65076	20699
Independent reflections	4846 [R_{int} = 0.0933, R_{sigma} = 0.0578]	2710 [R_{int} = 0.0413, R_{sigma} = 0.0252]
Data/restraints/parameters	4846/75/344	2710/0/143
Goodness-of-fit on F^2	1.175	1.203
Final R indexes [I>=2σ (I)]	R_1 = 0.0622, wR_2 = 0.1183	R_1 = 0.0311, wR_2 = 0.0691
Final R indexes [all data]	R_1 = 0.1036, wR_2 = 0.1311	R_1 = 0.0336, wR_2 = 0.0699
Largest diff. peak/hole / e Å^{-3}	1.59/-1.59	2.08/-1.83

4. Computations

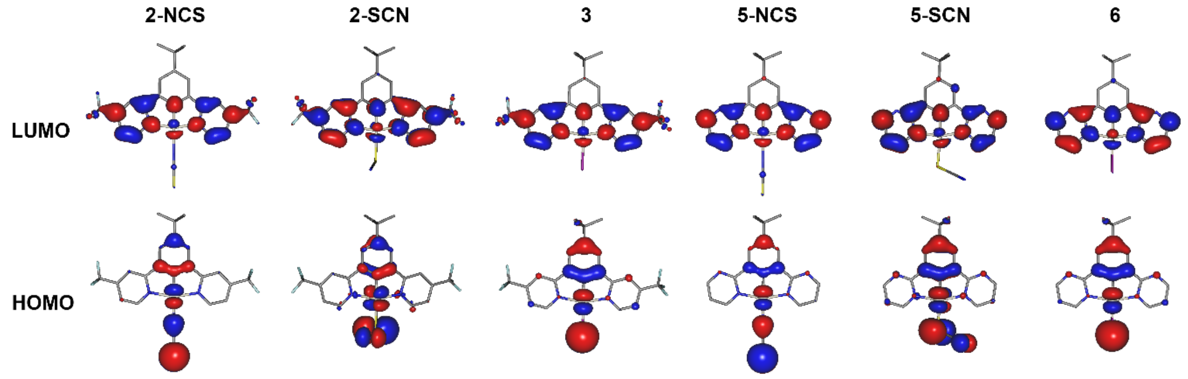

Figure S4.1. Frontier molecular orbital surface plots for complexes **2**, **3**, **5**, and **6** (including SCN/NCS isomers of **2** and **5**) at the S_0 geometry obtained at B3LYP/def2-SVP/CPCM(CH_2Cl_2) level of theory.

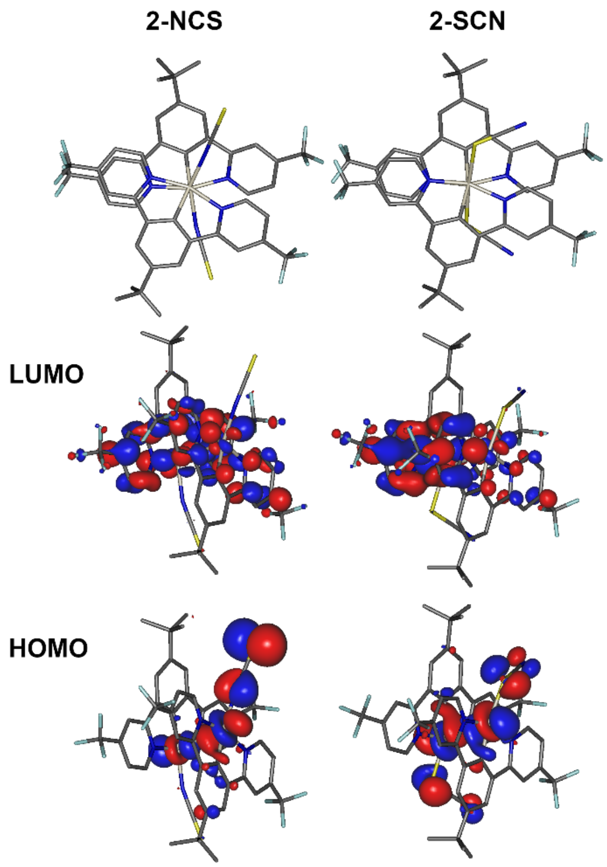

Figure S4.2. Structural geometry and frontier molecular orbital surface plots for isomeric SCN/NCS dimers/excimers of complex **2**. T_1 geometry obtained at BP86/def2-SVP level with subsequent single-point energy calculation using B3LYP/def2-SVP/CPCM(CH_2Cl_2) level of theory.

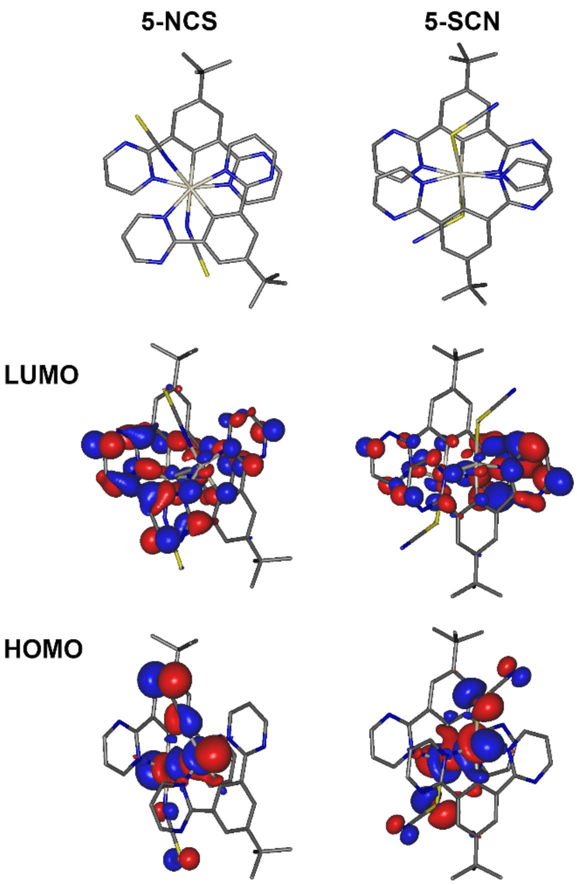

Figure S4.3. Structural geometry and frontier molecular orbital surface plots for isomeric SCN/NCS dimers/excimers of complex **5**. T_1 geometry obtained at BP86/def2-SVP level with subsequent single-point energy calculation using B3LYP/def2-SVP/CPCM(CH_2Cl_2) level of theory.

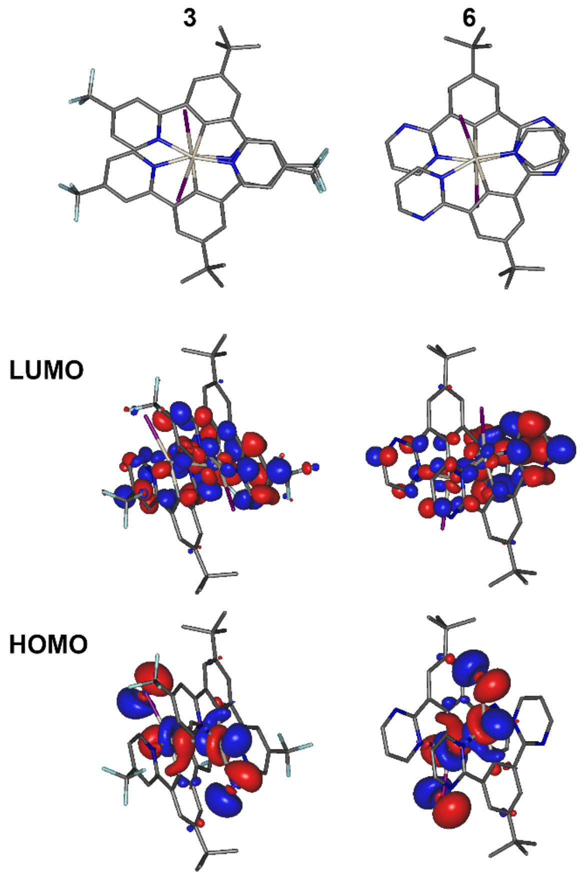

Figure S4.4. Structural geometry and frontier molecular orbital surface plots for dimers/excimers of complexes **3** and **6**. T_1 geometry obtained at BP86/def2-SVP level with subsequent single-point energy calculation using B3LYP/def2-SVP/CPCM(CH_2Cl_2) level of theory.

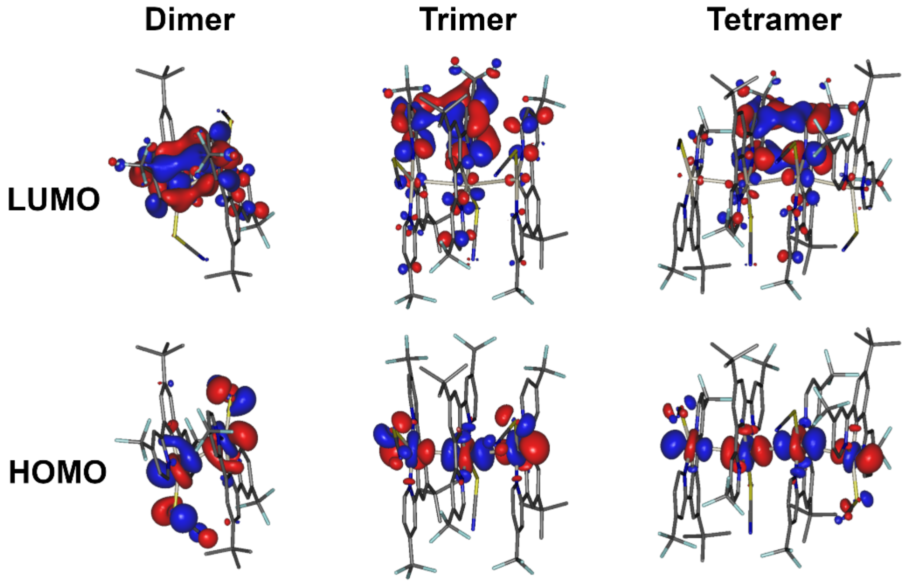

Figure S4.5. HOMO and LUMO iso surfaces in dimers and trimers of complex **2-SCN** at T_1 state geometry.

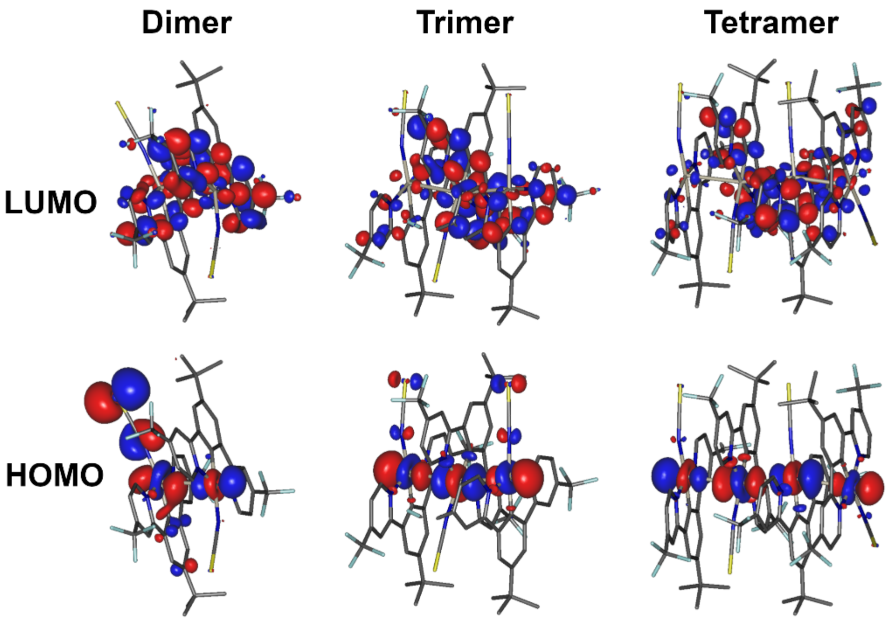

Figure S4.6. HOMO and LUMO iso surfaces in dimers and trimers of complex **2-NCS** at T_1 state geometry.

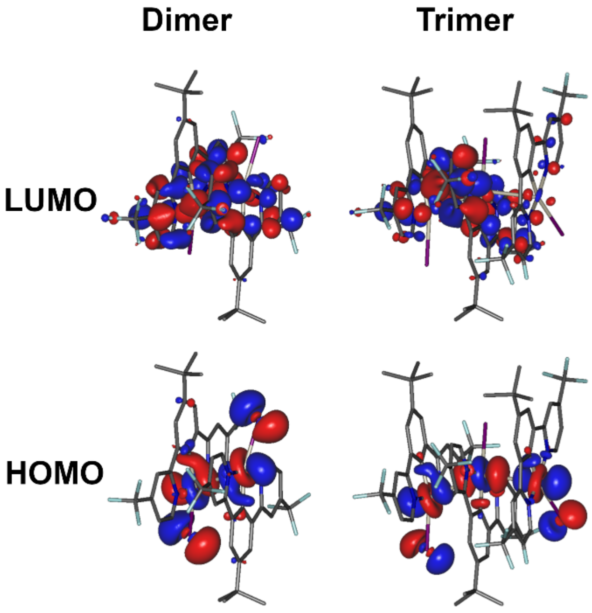

Figure S4.7. HOMO and LUMO iso surfaces in dimers and trimers of complex **3** at T_1 state geometry.

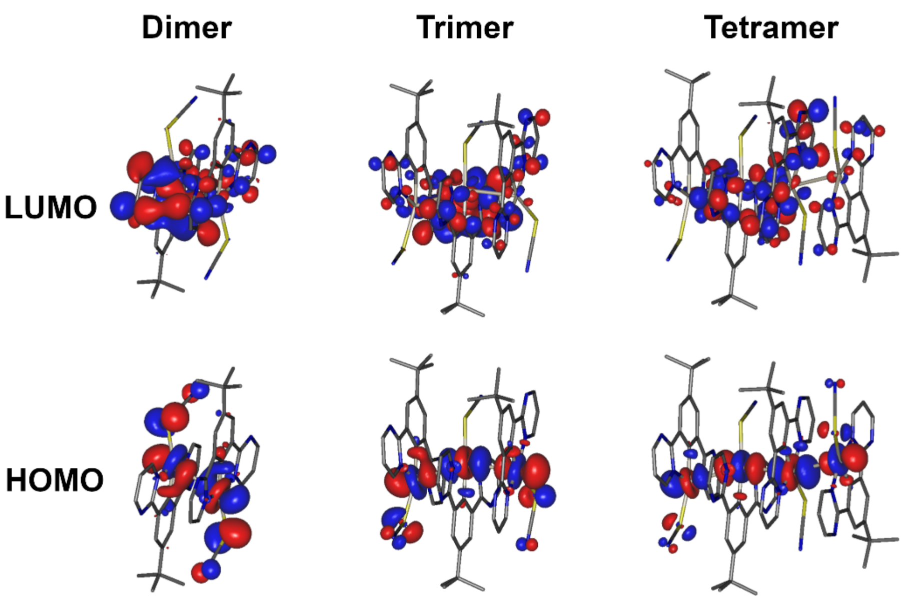

Figure S4.8. HOMO and LUMO iso surfaces in dimers and trimers of complex **5-SCN** at T_1 state geometry.

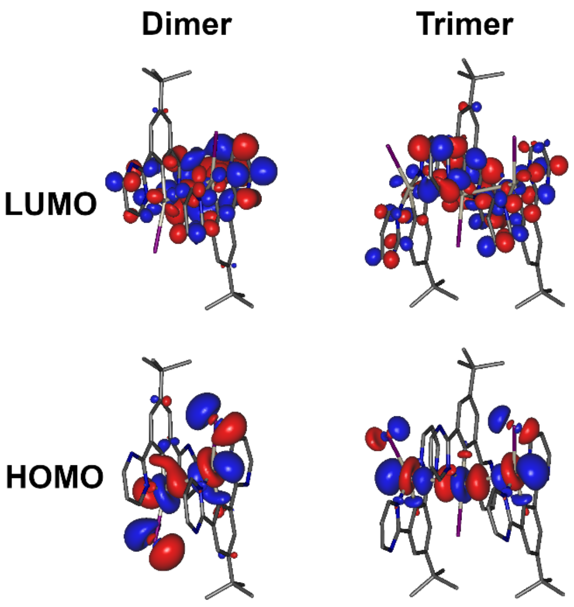

Figure S4.9. HOMO and LUMO iso surfaces in dimers and trimers of complex **6** at T_1 state geometry.

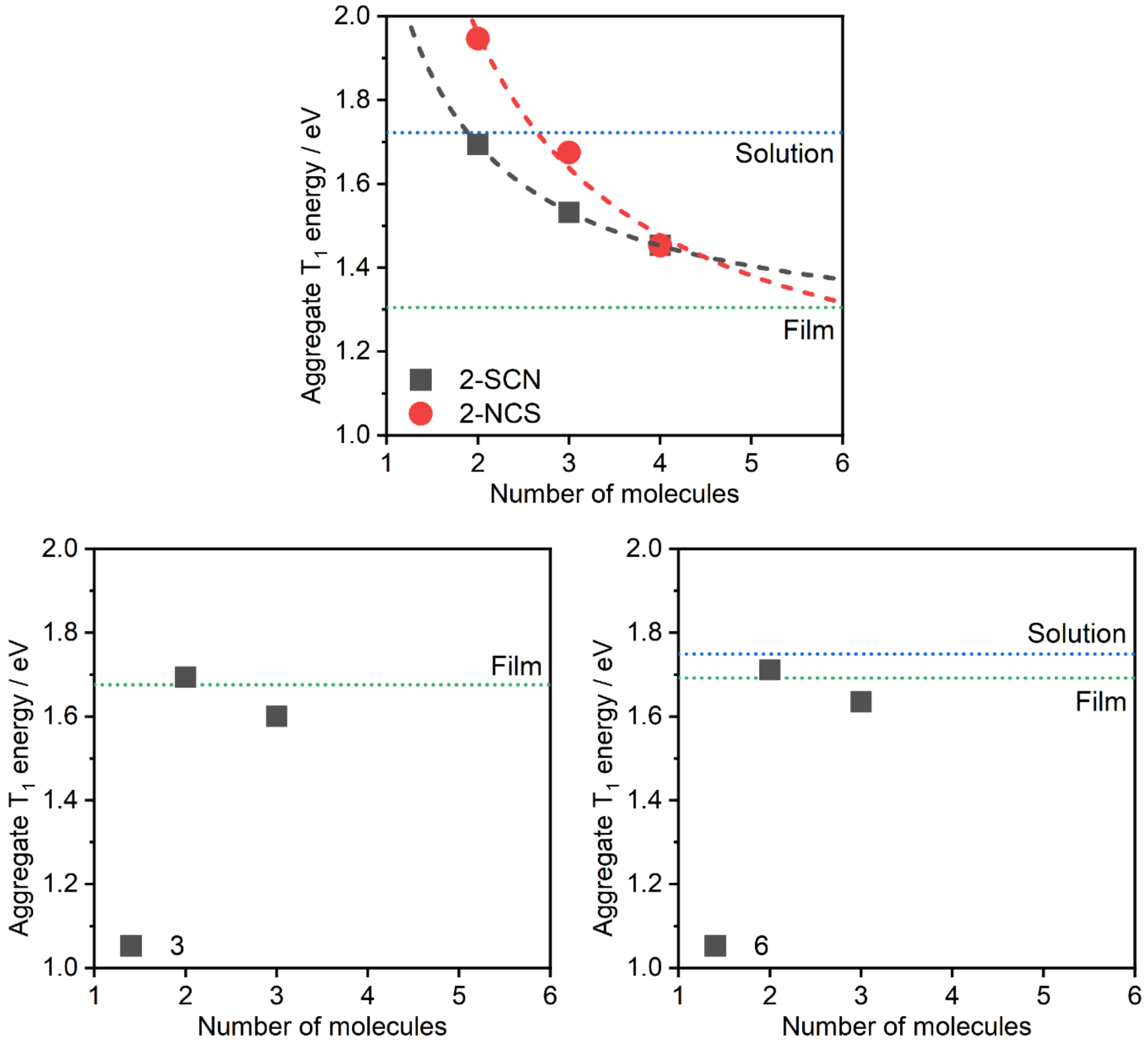

Figure S4.10. Predicted peak photoluminescence energy as a function of the number of molecules in the aggregate for complexes **2**, **3** and **6**. Dotted lines indicate the experimental peak energy in solution (excimer) or solid film (aggregate), while dashed lines represent the best fit to the equation $E(n) = a + b/n$, where n is the number of molecules.

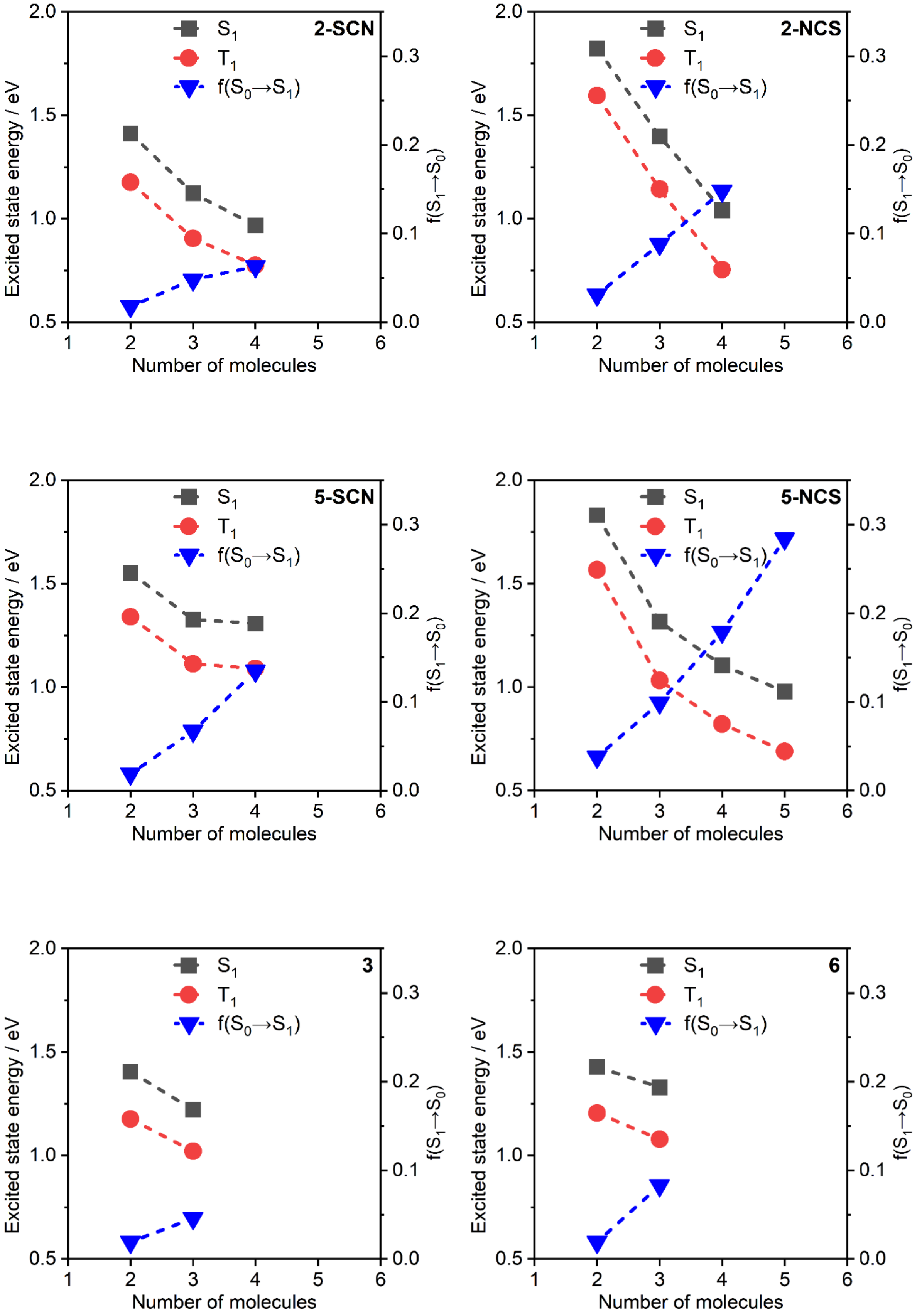

Figure S4.11. Dependence of the S_1 and T_1 energy and $S_1 \rightarrow S_0$ oscillator strength on aggregate size.

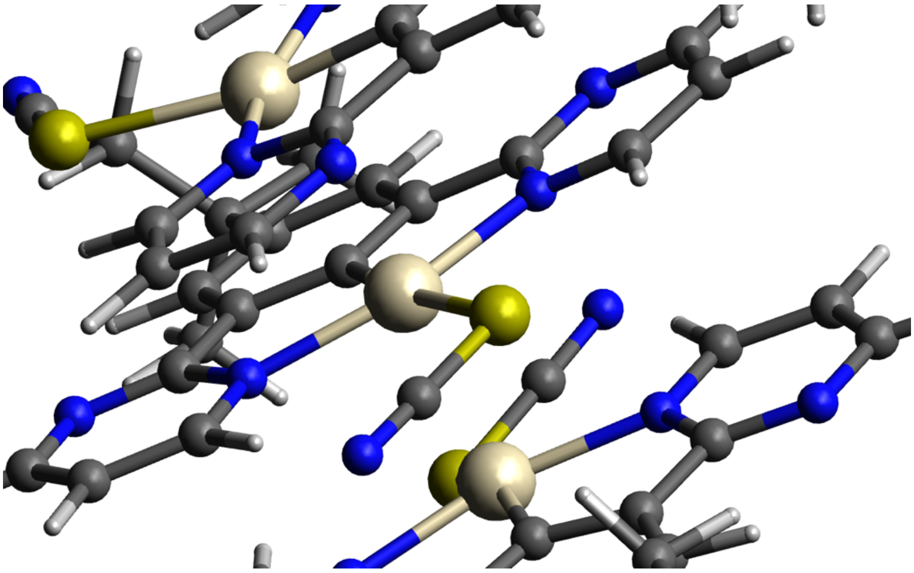

5-SCN trimer

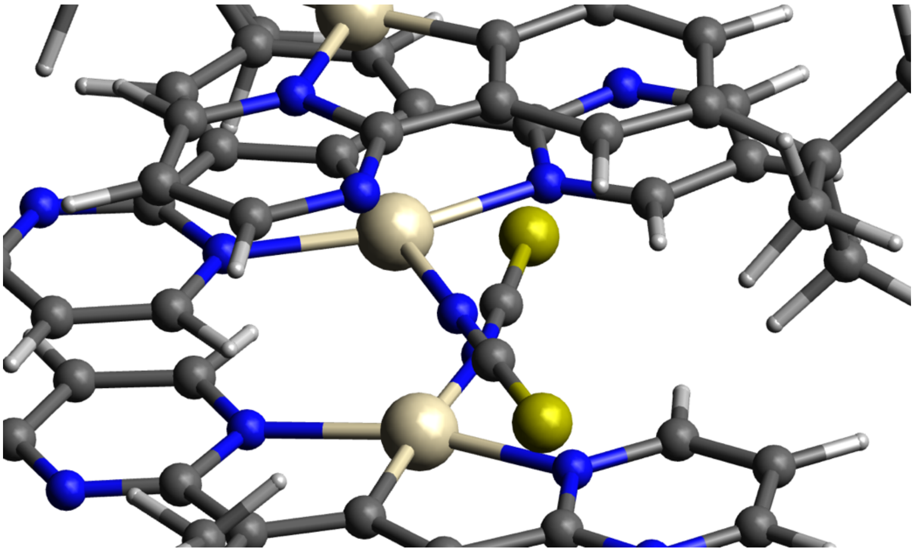

5-NCS trimer

Figure S4.12. Views of the relative dispositions of the NCS ion in neighbouring molecules, as obtained from the calculations of the trimers of the SCN (top) and NCS (bottom) forms of complex **5**. Note that the SCN ligands in the former adopt a roughly coplanar geometry with the Pt(N^C^N) fragment. That contrasts with the bent geometry in the crystal structure in Figure S3.1, suggesting that there may be little energetic difference between the conformations.

Table S4.1. Calculated properties of *anti* dimers of complexes **2**, **3**, **5**, and **6** at T_1 geometry using the BP86/def2-SVP level of theory for geometry optimisation. Single-point energy obtained at B3LYP/def2-SVP/CPCM(CH_2Cl_2).

Complex	Pt-Pt distance, Å	X-Pt-Pt-X dihedral, °	f($S_1 \rightarrow S_0$)	S_1, eV	T_1, eV
**2-SCN**	2.967	173.9	0.018	1.413	1.177
**2-NCS**	3.106	145.8	0.031	1.824	1.596
**3**	2.981	151.2	0.019	1.406	1.177
**5-SCN**	2.962	170.1	0.019	1.553	1.340
**5-NCS**	2.937	135.8	0.038	1.832	1.568
**6**	2.982	160.0	0.019	1.428	1.205

Table S4.2. Calculated properties of aggregates of complex **2** (isomer **2-NCS**) at T_1 geometry using the BP86/def2-SVP level of theory for geometry optimisation. Single-point energy obtained at B3LYP/def2-SVP/CPCM(CH_2Cl_2).

No of molecules	Pt-Pt distance range, Å	X-Pt-Pt-X dihedral range, °	f($S_1 \rightarrow S_0$)	S_1, eV	T_1, eV
**2**	3.106	145.8	0.031	1.824	1.596
**3**	3.003-3.019	128.9-141.5	0.088	1.399	1.144
**4**	2.919-3.063	125.2-145.3	0.148	1.042	0.755

Table S4.3. Calculated properties of aggregates of complex **2** (isomer **2-SCN**) at T_1 geometry using the BP86/def2-SVP level of theory for geometry optimisation. Single-point energy obtained at B3LYP/def2-SVP/CPCM(CH_2Cl_2).

No of molecules	Pt-Pt distance range, Å	X-Pt-Pt-X dihedral range, °	f($S_1 \rightarrow S_0$)	S_1, eV	T_1, eV
**2**	2.967	173.9	0.018	1.413	1.177
**3**	3.015-3.016	171.0-178.5	0.048	1.123	0.906
**4**	3.040-3.106	134.5-179.6	0.063	0.969	0.776

Table S4.4. Calculated properties of aggregates of complex **3** at T_1 geometry using the BP86/def2-SVP level of theory for geometry optimisation. Single-point energy obtained at B3LYP/def2-SVP/CPCM(CH_2Cl_2).

No of molecules	Pt-Pt distance range, Å	X-Pt-Pt-X dihedral range, °	$f(S_1 \rightarrow S_0)$	S_1, eV	T_1, eV
**2**	2.981	151.2	0.019	1.406	1.177
**3**	3.036-3.119	116.8-148.3	0.046	1.222	1.021

Table S4.5. Calculated properties of aggregates of complex **5** (isomer **5-NCS**) at T_1 geometry using the BP86/def2-SVP level of theory for geometry optimisation. Single-point energy obtained at B3LYP/def2-SVP/CPCM(CH_2Cl_2).

No of molecules	Pt-Pt distance range, Å	X-Pt-Pt-X dihedral range, °	$f(S_1 \rightarrow S_0)$	S_1, eV	T_1, eV
**2**	2.937	135.8	0.038	1.832	1.568
**3**	2.929-2.930	139.0-139.7	0.099	1.316	1.033
**4**	2.969-2.986	136.6-140.5	0.179	1.107	0.822
**5**	2.932-3.036	137.6-140.4	0.284	0.979	0.690

Table S4.6. Calculated properties of aggregates of complex **5** (isomer **5-SCN**) at T_1 geometry using the BP86/def2-SVP level of theory for geometry optimisation. Single-point energy obtained at B3LYP/def2-SVP/CPCM(CH_2Cl_2).

No of molecules	Pt-Pt distance range, Å	X-Pt-Pt-X dihedral range, °	$f(S_1 \rightarrow S_0)$	S_1, eV	T_1, eV
**2**	2.962	170.1	0.019	1.553	1.340
**3**	2.955-3.027	127.1-168.8	0.067	1.326	1.113
**4**	2.967-3.126	131.5-179.2	0.135	1.309	1.091

Table S4.7. Calculated properties of aggregates of complex **6** at T_1 geometry using the BP86/def2-SVP level of theory for geometry optimisation. Single-point energy obtained at B3LYP/def2-SVP/CPCM(CH_2Cl_2).

No of molecules	Pt-Pt distance range, Å	X-Pt-Pt-X dihedral range, °	$f(S_1 \rightarrow S_0)$	S_1, eV	T_1, eV
**2**	2.982	160.0	0.019	1.428	1.205
**3**	2.972-2.986	127.3-136.2	0.083	1.330	1.079

5. Photophysics

a) Solution state

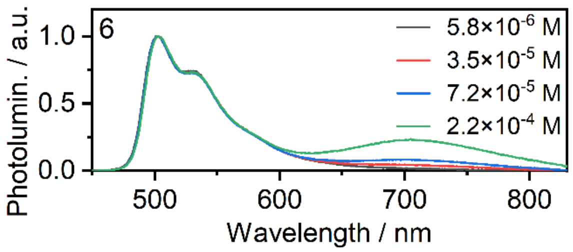

Figure S5.1. Normalised photoluminescence spectra of complex **6** in CH_2Cl_2 at the concentrations indicated in the figure legend.

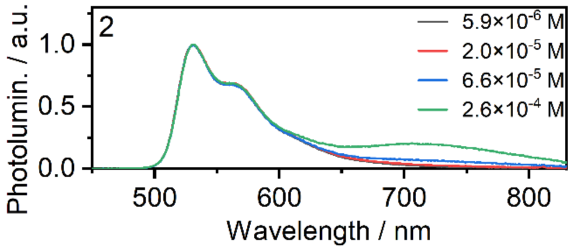

Figure S5.2. Normalised photoluminescence spectra of complex **2** in CH_2Cl_2 at the concentrations indicated in the figure legend.

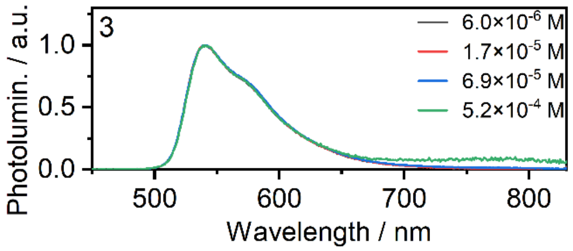

Figure S5.3. Normalised photoluminescence spectra of complex **2** in CH_2Cl_2 at the concentrations indicated in the figure legend.

b) Solid film

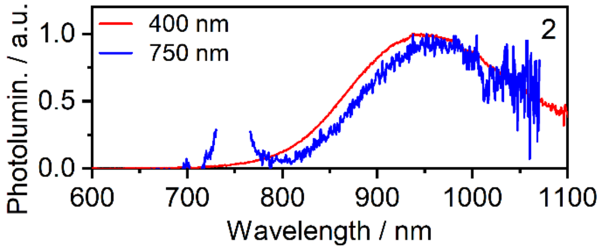

Figure S5.4.Normalised photoluminescence spectra of **2** in thermally evaporated neat film with long- and short-wavelength excitation.

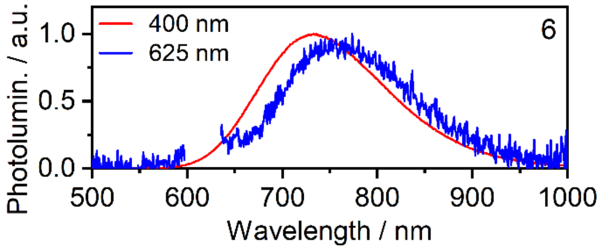

Figure S5.5.Normalised photoluminescence spectra of **6** in thermally evaporated neat film with long- and short-wavelength excitation.

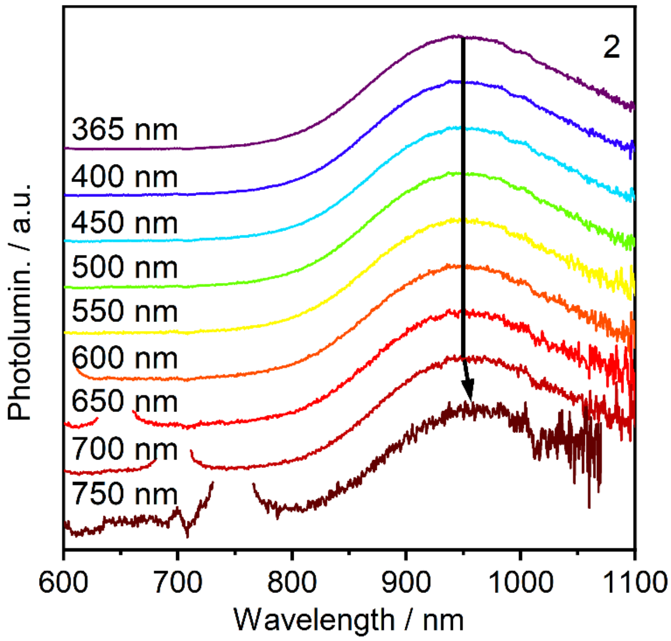

Figure S5.6.Normalised photoluminescence spectra of **2** in thermally evaporated neat film recorded with the excitation wavelengths indicated in the figure legend.

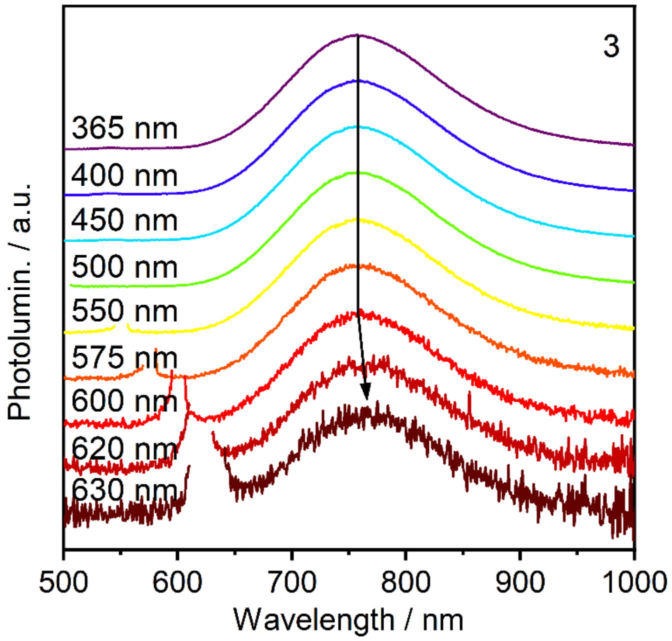

Figure S5.7.Normalised photoluminescence spectra of **3** in thermally evaporated neat film recorded with excitation wavelengths indicated in the figure legend.

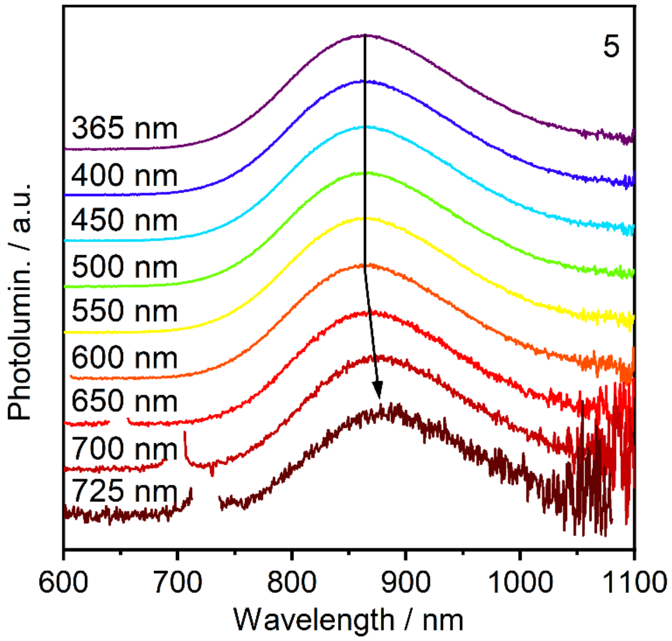

Figure S5.8.Normalised photoluminescence spectra of **5** in thermally evaporated neat film recorded with excitation wavelengths indicated in the figure legend.

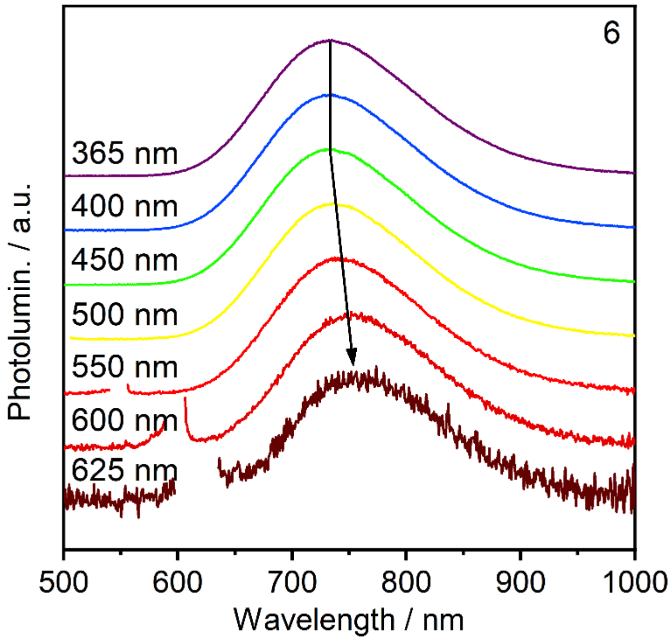

Figure S5.9.Normalised photoluminescence spectra of **6** in thermally evaporated neat film recorded with excitation wavelengths indicated in the figure legend.

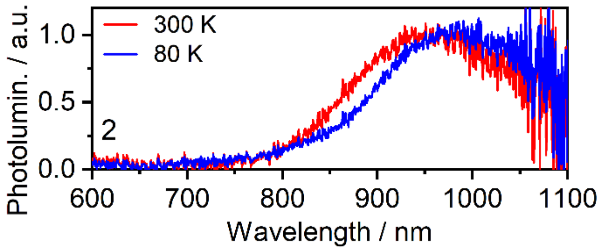

Figure S5.10.Normalised photoluminescence spectra of **2** in thermally evaporated neat film at 300 K and 80 K.

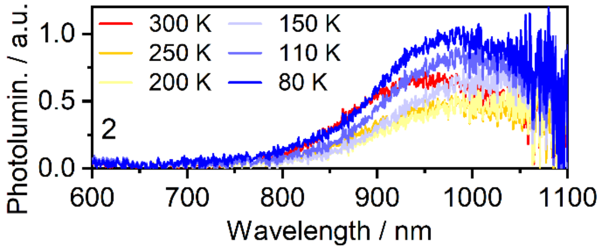

Figure S5.11.Photoluminescence spectra of **2** in thermally evaporated neat film at the temperatures indicated in the figure legend.

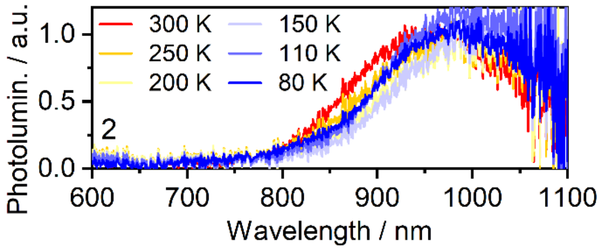

Figure S5.12.Normalised photoluminescence spectra of **2** in thermally evaporated neat film at the temperatures indicated in the figure legend.

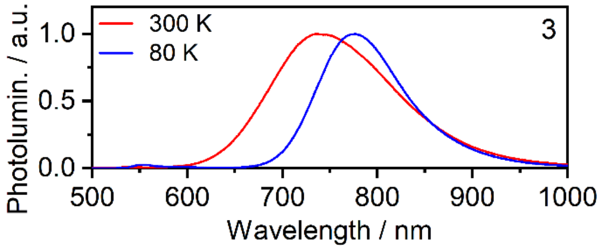

Figure S5.13.Normalised photoluminescence spectra of **3** in thermally evaporated neat film at 300 K and 80 K.

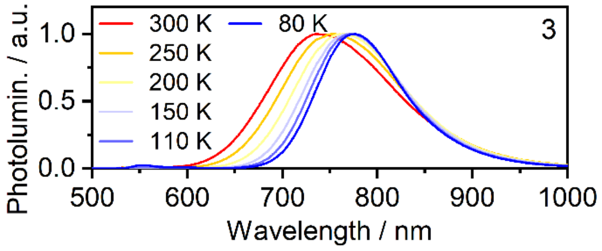

Figure S5.14.Normalised photoluminescence spectra of **3** in thermally evaporated neat film at the temperatures indicated in the figure legend.

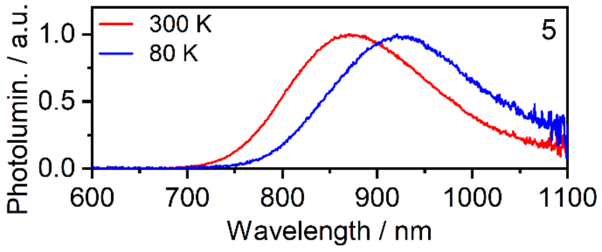

Figure S5.15.Normalised photoluminescence spectra of **5** in thermally evaporated neat film at 300 K and 80 K.

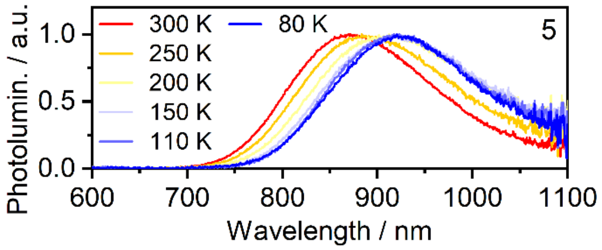

Figure S5.16.Normalised photoluminescence spectra of **5** in thermally evaporated neat film at the temperatures indicated in the figure legend.

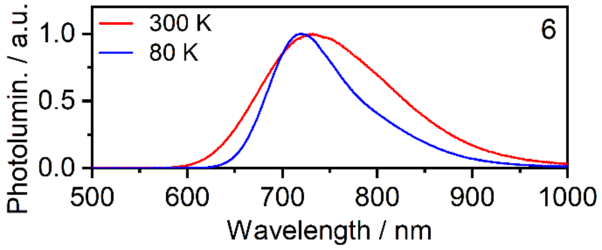

Figure S5.17.Normalised photoluminescence spectra of **6** in thermally evaporated neat film at 300 K and 80 K.

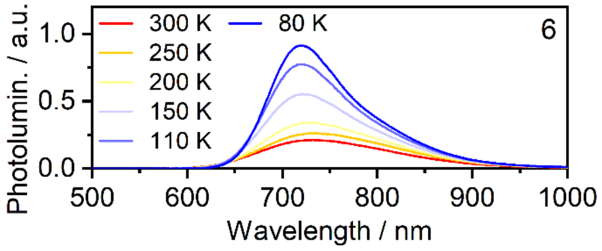

Figure S5.18.Photoluminescence spectra of **6** in thermally evaporated neat film at the temperatures indicated in the figure legend.

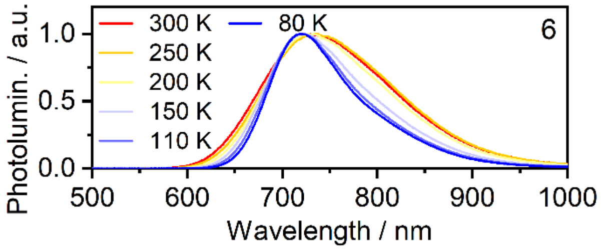

Figure S5.19.Normalised photoluminescence spectra of **6** in thermally evaporated neat film at temperatures indicated in the figure legend.

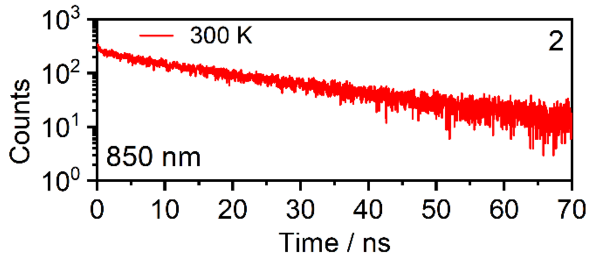

Figure S5.20. Photoluminescence decay trace of **2** in vacuum deposited neat film.

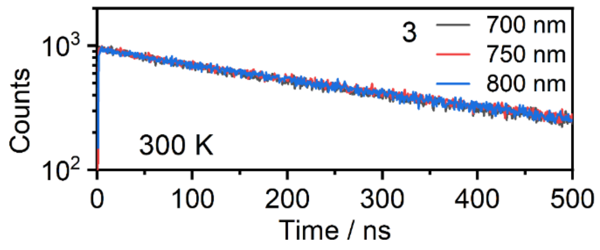

Figure S5.21. Photoluminescence decay traces of **3** in vacuum deposited neat film at 300 K and collection wavelengths indicated in the figure legend.

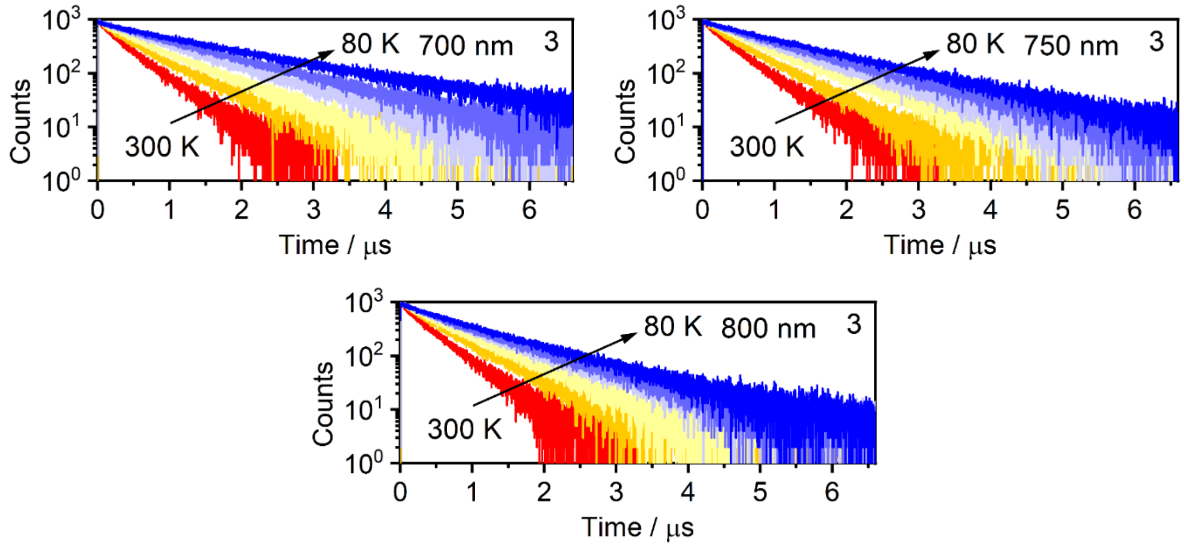

Figure S5.22. Photoluminescence decay traces of **3** in vacuum deposited neat film at temperatures from 300 K to 80 K and collection wavelengths indicated in the figure legend.

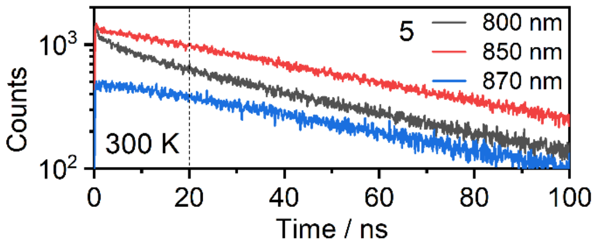

Figure S5.23. Photoluminescence decay traces of **5** in vacuum deposited neat film at 300 K and collection wavelengths indicated in figure legend. The vertical dashed line divides the decay into two regimes with divergent behaviour: luminescence quenching at shorter wavelengths and luminescence build-up at longer (*on the left*) and monoexponential luminescence decay (*on the right*).

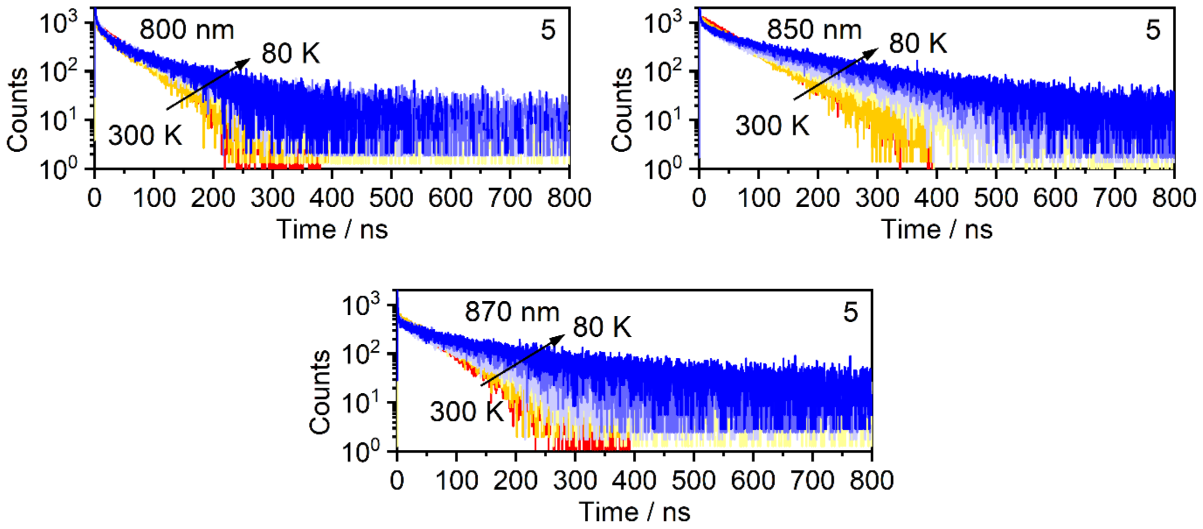

Figure S5.24. Photoluminescence decay traces of **5** in vacuum deposited neat film at temperatures from 300 K to 80 K and collection wavelengths indicated in the figure legend.

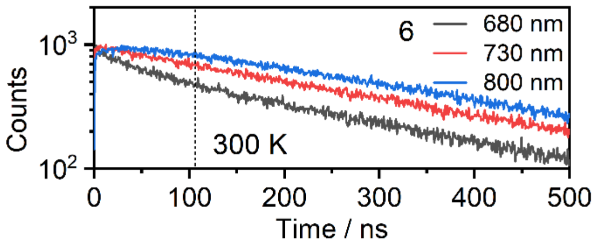

Figure S5.25. Photoluminescence decay traces of **6** in vacuum deposited neat film at 300 K and collection wavelengths indicated in figure legend. The vertical dashed line divides the decay into two regimes with divergent behaviour: luminescence quenching at shorter wavelengths and luminescence build-up at longer (*on the left*) and monoexponential luminescence decay (*on the right*).

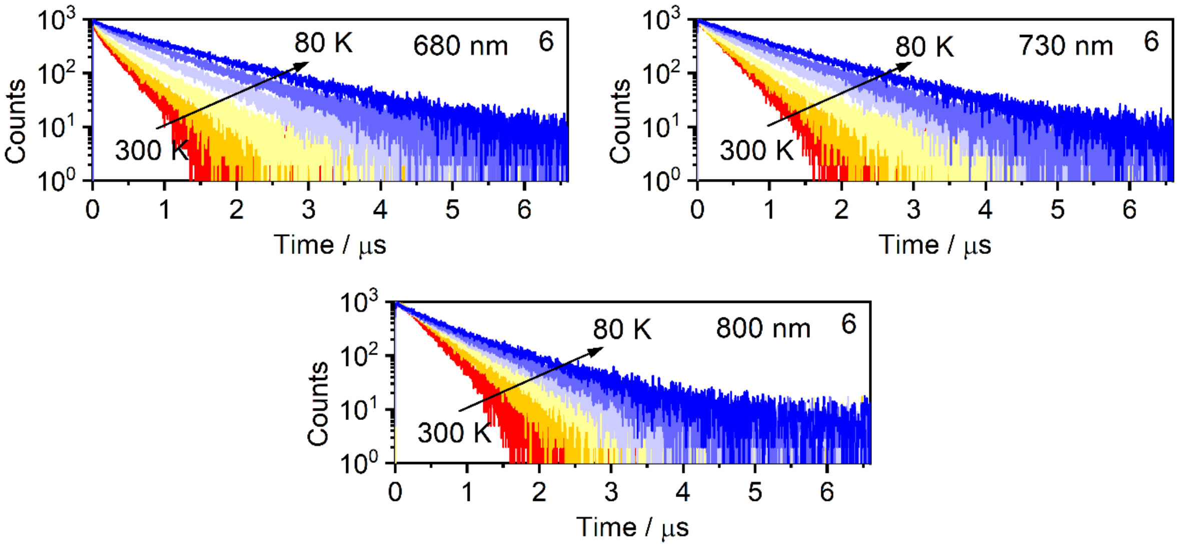

Figure S5.26. Photoluminescence decay traces of **6** in vacuum deposited neat film at temperatures from 300 K to 80 K and collection wavelengths indicated in the figure legend.

Table S5.1. Photoluminescence decay lifetime of complexes **2** in vacuum-deposited films.

**T, K**	**2** (850 nm)
	**τ_1, ns**
300	22

Table S5.2. Photoluminescence decay lifetime of complexes **3** in vacuum-deposited films.

**T, K**	**3** (750 nm)
	**τ_1, μs**
300	0.44
250	0.60
200	0.79
150	0.97
110	1.16
80	1.40

Table S5.3. Photoluminescence decay lifetime of complexes **5** in vacuum-deposited films.

**T, K**	**5** (870 nm)
	**τ_1, ns**
300	62
250	66
200	84
150	92
110	110
80	170

Table S5.4. Photoluminescence decay lifetime of complexes **6** in vacuum-deposited films.

**T, K**	**6** (730 nm)
	**τ_1, μs**
300	0.32
250	0.38
200	0.51
150	0.73
110	0.90
80	1.1

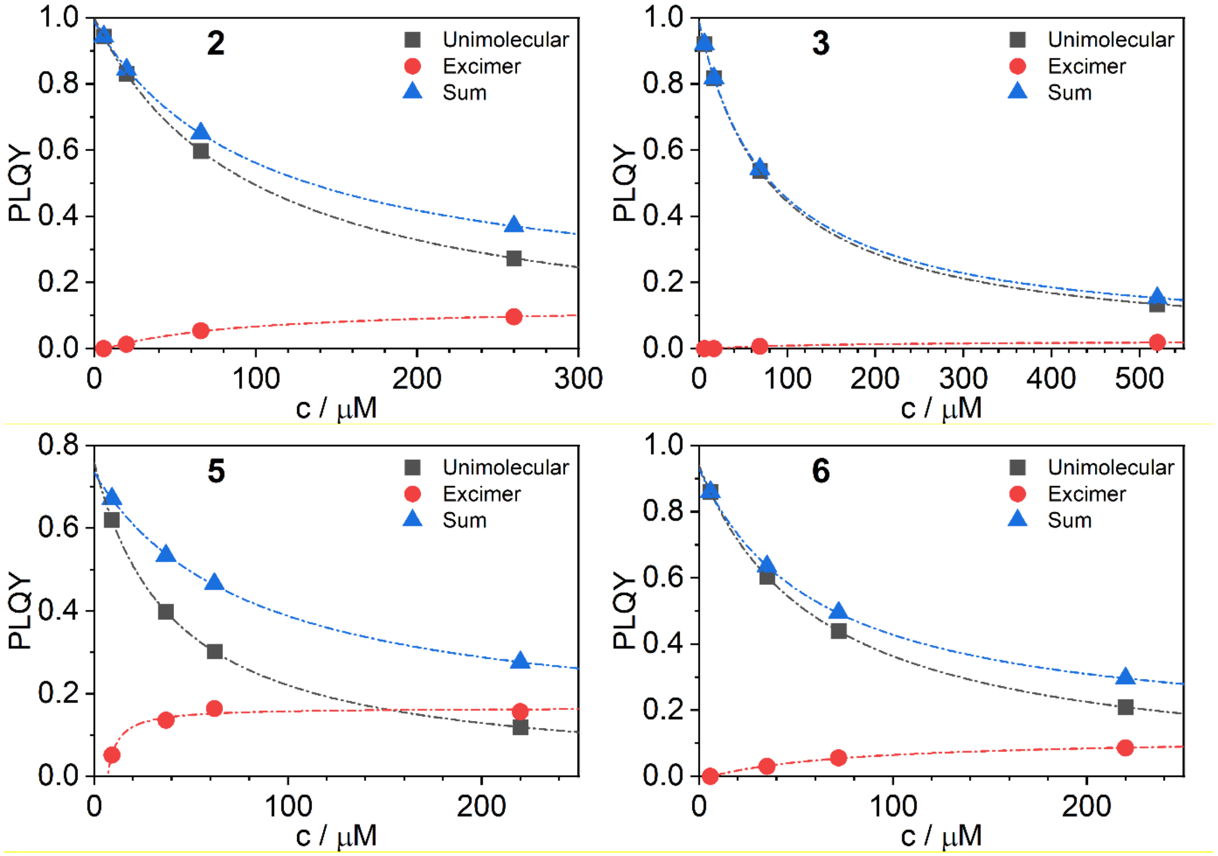

Figure S5.27. Photoluminescence quantum yield as a function of concentration in dichloromethane for complexes **2**, **3**, **5**, and **6**. Experimental data points are depicted as black squares (isolated molecules), red circles (excimers), and blue triangles (sum of the two). The graph shows contributions of each component to the total PLQY and their sum. The dashed and dotted lines serve as an eye guide and were created by fitting an equation of the form $y = \frac{a}{x+b} + c$ to the experimental data points, where: y – PLQY; x – concentration c, mol dm^{-3}; a, b, c – constants.

6. Electrochemistry

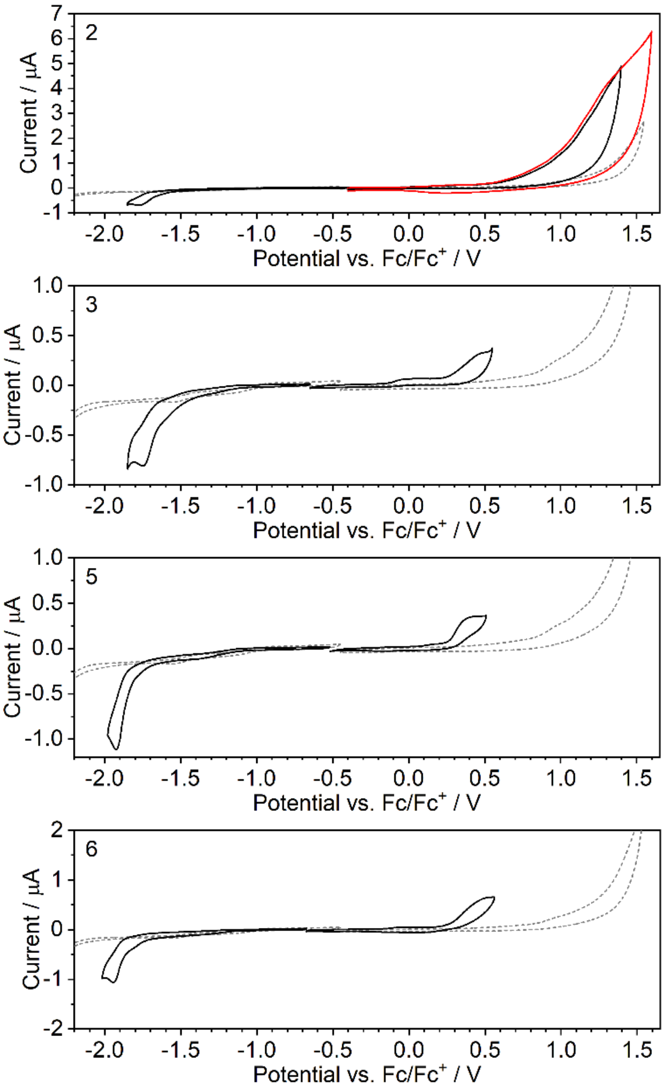

Figure S6.1 Cyclic voltammograms (CV) of complexes **2**, **3**, **5**, and **4** at c ~ 5×10^{-4} M in 0.1 M Bu_4NBF_4 / CH_2Cl_2 solution. *Black line*: first oxidation and reduction cycles; *red line*: oxidation cycle recorded at higher potential; *grey dashed line*: electrolyte background current.

Table S6.1. Electrochemical onset redox potentials of complexes **2**, **3**, **5**, and **4** recorded in 0.1 M Bu_4NBF_4 / CH_2Cl_2 solution at c = 10^{-3} M.

**Complex**	**E_{ox} / V**	**E_{red} / V**	**HOMO / eV**	**LUMO / eV**
**2**	~0.3*	-1.63	~5.4*	3.47
**3**	0.26	-1.63	5.36	3.47
**5**	0.27	-1.82	5.37	3.28
**6**	0.25	-1.85	5.35	3.25

* Oxidation onset potential for complex **2** cannot be precisely estimated due to the character of the oxidation process.

7. OLED devices

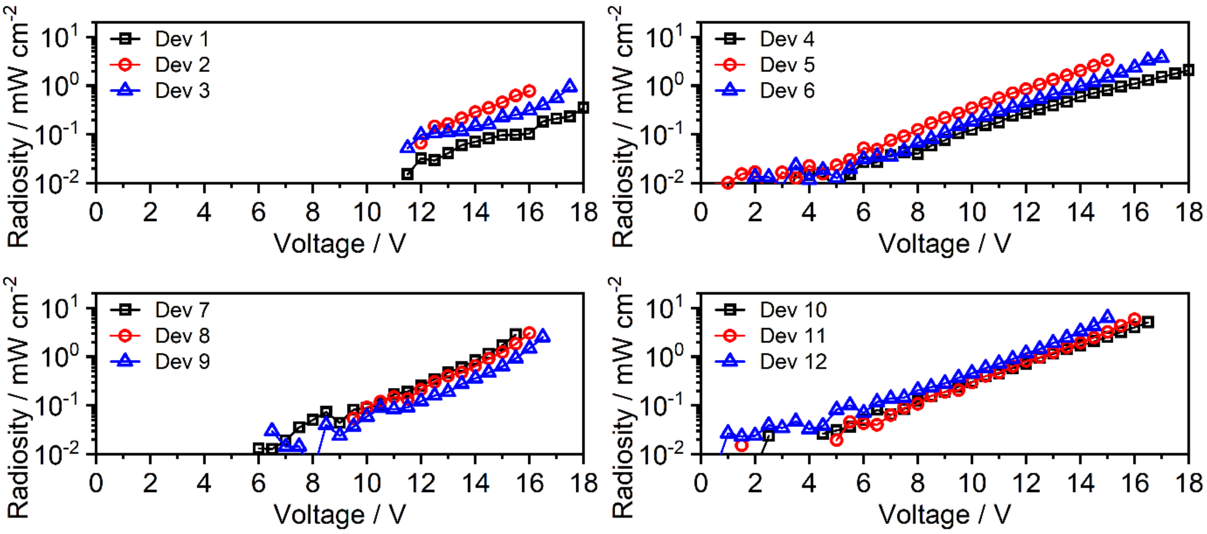

Figure S7.1. Radiosity-voltage characteristics of Devices 1-12.

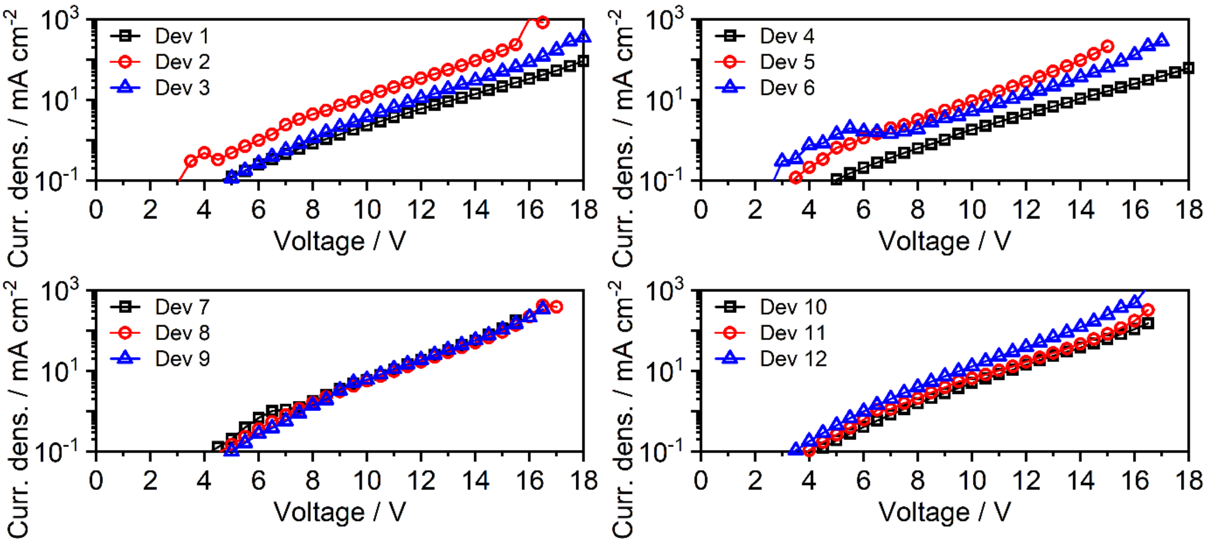

Figure S7.2. Current density-voltage (J-V) characteristics of Devices 1-6.

Table S7.1 Compilation of reported NIR OLED efficiency (λ_{el} ≈ 700-1000 nm) of platinum(II) complexes, other metal complexes and metal-free emitters.

Emitter type	λ_{el}, nm	EQE, %		Reference
		Max.	at 100 mA cm^{-2}	
Mono-Pt(II)	896	3.8	0.6	[25]
Mono-Pt(II)	771	2.1	*	[26]
Mono-Pt(II)	773	8.0	*	[26]
Mono-Pt(II)	898	0.75	*	[26]
Mono-Pt(II)	900	3.8	*	[26]
Mono-Pt(II)	1005	0.2	*	[26]
Di-Pt(II)	731	3.6	0.8	[27]
Di-Pt(II)	746	0.58	0.53	[28]
Aggregate Pt(II)	724	16.7	5.0	[29]
Aggregate Pt(II)	740	24	*	[30]
Aggregate Pt(II)	900	1.7	1.5	[31]
Aggregate Pt(II)	890	2.1	2.0	[31]
Aggregate Pt(II)	930	2.0	1.8	[31]
Aggregate Pt(II)	692	6.3	3.5	[32]
Aggregate Pt(II)	700	8.1	5.2	[32]
Aggregate Pt(II)	704	8.9	5.2	[32]
Aggregate Pt(II)	716	5.1	4.0	[33]
Mono-Os(II)	814	1.5	1.2	[34]
Mono-Os(II)	739	5.2	4.1	[35]
Mono-Os(II)	776	3.96	2.67	[36]
Mono-Os(II)	746	4.19	2.83	[36]
Mono-Os(II)	794	2.94	2.34	[36]
Mono-Os(II)	816	1.20	1.00	[36]
Mono-Ir(III)	760	4.5	3.5	[37]
Mono-Ir(III)	811	0.5	0.5	[37]
Mono-Ir(III)	780	2.2	1.9	[38]
Metal-free	786	0.77	0.28	[39]
Metal-free	802	0.43	0.43	[40]
Metal-free	864	0.20	0.20	[40]
Metal-free	840	1.15	0.85	[41]
Metal-free	904	0.02	0.016	[42]

* No data.

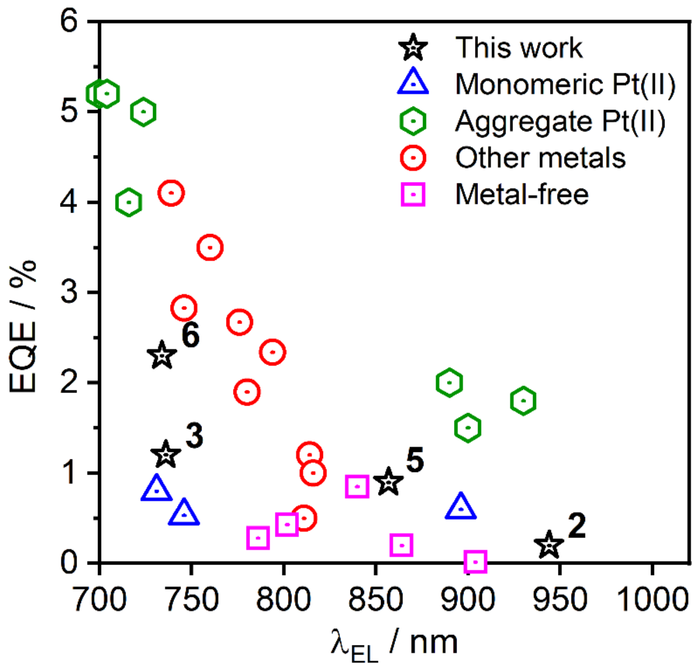

Figure S7.3. External quantum efficiency (EQE) at J = 100 mA cm^{-2} and electroluminescence spectrum maxima (λ_{EL}) of sample NIR OLEDs from the literature that use platinum(II) complexes or others as emitters indicated in the figure legend. See **Table S7.1** in the ESI for references.

8. Influence of ancillary ligand - summary

Table S8.1. Comparison of photophysical and electroluminescent properties of complexes **1-6** of the form Pt(N^C^N)-X.

**Cyclometallating ligand (N^C^N)**	**Auxiliary ligand (X)**	**Complex**	**Dilute CH_2Cl_2 solutions**			**Neat films**		**Non-doped OLED** [a]	
			**λ_{em}, nm**	**λ_{em}, nm (excimer)**	**Φ_{PL}**	**λ_{em}, nm**	**Φ_{PL}**	**λ_{el}, nm**	**EQE, %**
**1,3-bis(4-trifluoromethyl-pyrid-2-yl)-5-*t*-butylbenzene**	Cl	**1**	534	744	0.91	753	0.02	765	0.8
	SCN	**2**	531	720	1.0	950	~0.01	944	0.3
	I	**3**	541	~775	0.92	740	0.09	735	2.5
**1,3-bis(pyrimidin-2-yl)-5-*t*-butylbenzene**	Cl	**4**	500	701	0.49	800	0.03	817	1.2
	SCN	**5**	497	659	0.67	872	0.02	857	1.1
	I	**6**	502	709	0.87	733	0.12	734	4.2

[a] We selected OLEDs with representative emissive layer thickness of 2 nm.

9. References

[1] F. Neese, *WIREs Comput. Mol. Sci.* **2018**, *8:e1327*, 1.

[2] F. Neese, *WIREs Comput. Mol. Sci.* **2012**, *2*, 73.

[3] S. Lehtola, C. Steigemann, M. J. T. Oliveira, M. A. L. Marques, *SoftwareX* **2018**, *7*, 1.

[4] A.-R. Allouche, *J. Comput. Chem.* **2011**, *32*, 174.

[5] Avogadro: an open-source molecular builder and visualization tool. Version 1.2.0, https://avogadro.cc.

[6] M. D. Hanwell, D. E. Curtis, D. C. Lonie, T. Vandermeersch, E. Zurek, G. R. Hutchison, *J. Cheminform.* **2012**, *4*, 17.

[7] A. D. Becke, *J. Chem. Phys.* **1993**, *98*, 5648.

[8] P. J. Stephens, F. J. Devlin, C. F. Chabalowski, M. J. Frisch, *J. Phys. Chem.* **1994**, *98*, 11623.

[9] F. Weigend, R. Ahlrichs, *Phys. Chem. Chem. Phys.* **2005**, *7*, 3297.

[10] A. D. Becke, *Phys. Rev. A* **1988**, *38*, 3098.

[11] A. Hellweg, C. Hättig, S. Höfener, W. Klopper, *Theor. Chem. Acc.* **2007**, *117*, 587.

[12] F. Weigend, *Phys. Chem. Chem. Phys.* **2006**, *8*, 1057.

[13] F. Neese, F. Wennmohs, A. Hansen, U. Becker, *Chem. Phys.* **2009**, *356*, 98.

[14] R. Izsák, F. Neese, *J. Chem. Phys.* **2011**, *135*, 144105.

[15] S. Grimme, S. Ehrlich, L. Goerigk, *J. Comput. Chem.* **2011**, *32*, 1456.

[16] S. Grimme, J. Antony, S. Ehrlich, H. Krieg, *J. Chem. Phys.* **2010**, *132*, 154104.

[17] P. Data, P. Pander, M. Lapkowski, A. Swist, J. Soloducho, R. R. Reghu, J. V. Grazulevicius, *Electrochim. Acta* **2014**, *128*, 430.

[18] P. Pander, P. Data, R. Turczyn, M. Lapkowski, A. Swist, J. Soloducho, A. P. Monkman, *Electrochim. Acta* **2016**, *210*, 773.

[19] C. M. Cardona, W. Li, A. E. Kaifer, D. Stockdale, G. C. Bazan, *Adv. Mater.* **2011**, *23*, 2367.

[20] J.-L. Bredas, *Mater. Horiz.* **2014**, *1*, 17.

[21] D. de Sa Pereira, A. P. Monkman, P. Data, *J. Vis. Exp.* **2018**, DOI: 10.3791/56593.

[22] P. H. Pander, A. Sil, R. Salthouse, C. Harris, M. Walden, D. S. Yufit, G. Williams, F. B. Dias, *J. Mater. Chem. C* **2022**, DOI: 10.1039/D2TC01511K.

[23] O. V. Dolomanov, L. J. Bourhis, R. J. Gildea, J. A. K. Howard, H. Puschmann, *J. Appl. Crystallogr.* **2009**, *42*, 339.

[24] G. M. Sheldrick, *Acta Crystallogr. Sect. A Found. Crystallogr.* **2008**, *64*, 112.

[25] J. R. Sommer, R. T. Farley, K. R. Graham, Y. Yang, J. R. Reynolds, J. Xue, K. S. Schanze, *ACS Appl. Mater. Interfaces* **2009**, *1*, 274.

[26] K. R. Graham, Y. Yang, J. R. Sommer, A. H. Shelton, K. S. Schanze, J. Xue, J. R. Reynolds, *Chem. Mater.* **2011**, *23*, 5305.

[27] M. Z. Shafikov, P. Pander, A. V. Zaytsev, R. Daniels, R. Martinscroft, F. B. Dias, J. A. G. Williams, V. N. Kozhevnikov, *J. Mater. Chem. C* **2021**, *9*, 127.

[28] K. Zhang, Y. Liu, Z. Hao, G. Lei, S. Cui, W. Zhu, Y. Liu, *Org. Electron.* **2020**, *87*, 105902.

[29] X. Yang, H. Guo, X. Xu, Y. Sun, G. Zhou, W. Ma, Z. Wu, *Adv. Sci.* **2019**, *6*, 1801930.

[30] K. Tuong Ly, R. W. Chen-Cheng, H. W. Lin, Y. J. Shiau, S. H. Liu, P. T. Chou, C. S. Tsao, Y. C. Huang, Y. Chi, *Nat. Photonics* **2017**, *11*, 63.

[31] Y.-C. Wei, S. F. Wang, Y. Hu, L.-S. Liao, D.-G. Chen, K.-H. Chang, C.-W. Wang, S.-H. Liu, W.-H. Chan, J.-L. Liao, W.-Y. Hung, T.-H. Wang, P.-T. Chen, H.-F. Hsu, Y. Chi, P.-T. Chou, *Nat. Photonics* **2020**, *14*, 570.

[32] W. Xiong, F. Meng, H. Tan, Y. Wang, P. Wang, Y. Zhang, Q. Tao, S. Su, W. Zhu, *J. Mater. Chem. C* **2016**, *4*, 6007.

[33] X. Wu, D.-G. Chen, D. Liu, S.-H. Liu, S.-W. Shen, C.-I. Wu, G. Xie, J. Zhou, Z.-X. Huang, C.-Y. Huang, S.-J. Su, W. Zhu, P.-T. Chou, *J. Am. Chem. Soc.* **2020**, *142*, 7469.

[34] T.-C. Lee, J. Hung, Y. Chi, Y. Cheng, G. Lee, P. Chou, C. Chen, C. Chang, C. Wu, *Adv. Funct. Mater.* **2009**, *19*, 2639.

[35] Y. Yuan, J. L. Liao, S. F. Ni, A. K. Y. Jen, C. S. Lee, Y. Chi, *Adv. Funct. Mater.* **2020**, *30*, 1.

[36] X. Peng, C. Yeh, S. F. Wang, J. Yan, S. Gan, S. Su, X. Zhou, Y. Zhang, Y. Chi, *Adv. Opt. Mater.* **2022**, *2201291*, 2201291.

[37] J. Xue, L. Xin, J. Hou, L. Duan, R. Wang, Y. Wei, J. Qiao, *Chem. Mater.* **2017**, *29*, 4775.

[38] R. Tao, J. Qiao, G. Zhang, L. Duan, C. Chen, L. Wang, Y. Qiu, *J. Mater. Chem. C* **2013**, *1*, 6446.

[39] T. Liu, G. Xie, C. Zhong, S. Gong, C. Yang, *Adv. Funct. Mater.* **2018**, *28*, 1706088.

[40] X. Du, J. Qi, Z. Zhang, D. Ma, Z. Y. Wang, *Chem. Mater.* **2012**, *24*, 2178.

[41] A. Minotto, P. Murto, Z. Genene, A. Zampetti, G. Carnicella, W. Mammo, M. R. Andersson, E. Wang, F. Cacialli, *Adv. Mater.* **2018**, *30*, 1706584.

[42] D. G. Congrave, B. H. Drummond, P. J. Conaghan, H. Francis, S. T. E. Jones, C. P. Grey, N. C. Greenham, D. Credgington, H. Bronstein, *J. Am. Chem. Soc.* **2019**, *141*, 18390.
